# ACSS3 in brown fat drives propionate catabolism and its deficiency leads to autophagy and systemic metabolic dysfunction

**DOI:** 10.1002/ctm2.665

**Published:** 2022-02-20

**Authors:** Zhihao Jia, Xiyue Chen, Jingjuan Chen, Lijia Zhang, Stephanie N. Oprescu, Nanjian Luo, Yan Xiong, Feng Yue, Shihuan Kuang

**Affiliations:** ^1^ Department of Animal Sciences Purdue University West Lafayette Indiana; ^2^ Department of Biological Sciences Purdue University West Lafayette Indiana; ^3^ Center for Cancer Research Purdue University West Lafayette Indiana

**Keywords:** acyl‐CoA synthetase, adipose tissue, brown adipocytes, hydroxychloroquine, obesity, short‐chain fatty acid

## Abstract

Propionate is a gut microbial metabolite that has been reported to have controversial effects on metabolic health. Here we show that propionate is activated by acyl‐CoA synthetase short‐chain family member 3 (ACSS3), located on the mitochondrial inner membrane in brown adipocytes. Knockout of *Acss3* gene (Acss3^–/–^) in mice reduces brown adipose tissue (BAT) mass but increases white adipose tissue (WAT) mass, leading to glucose intolerance and insulin resistance that are exacerbated by high‐fat diet (HFD). Intriguingly, Acss3^–/–^ or HFD feeding significantly elevates propionate levels in BAT and serum, and propionate supplementation induces autophagy in cultured brown and white adipocytes. The elevated levels of propionate in Acss3^–/–^ mice similarly drive adipocyte autophagy, and pharmacological inhibition of autophagy using hydroxychloroquine ameliorates obesity, hepatic steatosis and insulin resistance of the Acss3^–/–^ mice. These results establish ACSS3 as the key enzyme for propionate metabolism and demonstrate that accumulation of propionate promotes obesity and Type 2 diabetes through triggering adipocyte autophagy.

## Graphical abstract

1. Acss3 is highly expressed in BAT and Acss3 knockout inhibits BAT adipogenesis, leading to smaller brown adipose tissue in vivo.
2. Loss of Acss3 causes elevated circulating propionate that promotes lipid accumulation and autophagy in adipose tissue.
3. Pharmacological inhibition of autophagy using hydroxychloroquine ameliorates obesity, hepatic steatosis and insulin resistance of the Acss3^–/–^ mice.

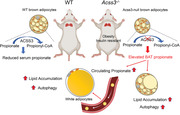

## 1 INTRODUCTION

The increasing prevalence of obesity and its associated metabolic diseases have posed formidable threats to human health.^1^, ^2^ Plasma lipid levels, especially free fatty acids (FAs) levels, are elevated in obesity and contribute to insulin resistance.^3^, ^4^ Thus, understanding the molecular mechanisms that promote utilisation and metabolism of FAs is imperative for development of new strategies to overcome obesity. FAs play many essential roles in living organisms as building blocks of bioactive lipids and energy source.^5^ Activation of FAs, which is the initial step of all metabolic processes of FAs, is catalysed by ACS family enzymes via replacing the terminal carboxyl group and forming a thioester with CoA.^6^ Due to the diversity of FAs in chain length and saturation, multiple enzymes with ACS activity are found to facilitate the activation of FAs.^7^ There are 26 ACSs in humans that are classified based on the carbon (C) chain length of their substrates.^5^, ^8^ ACSSs activate short‐chain fatty acids (SCFAs) of five carbons or shorter, of which acetate (C2), propionate (C3) and butyrate (C4) are the most abundant (≥95%).^5^

Emerging evidence has demonstrated that SCFAs produced by gut microbiota participate in many aspects of intracellular signalling and cellular metabolism, thus exerting vital roles in host metabolic health.^9^, ^10^, ^11^, ^12^, ^13^ To date, three ACSS members (ACSS1–3) have been identified in mammals, with ACSS1 and ACSS2 serving as the main enzymes to produce acetyl coenzyme A (acetyl‐CoA) from free acetate in mitochondria and cytoplasm.^14^ Acetyl‐CoA subsequently participates in the regulation of bioenergetics, cell proliferation and gene expression.^15^ ACSS3 is predicted to be a mitochondrial protein based on its deduced amino acid sequence.^16^ However, its substrate specificity, intramitochondrial localisation, tissue distribution and biological functions are poorly understood. The few studies available in the literature suggest that ACSS3 is a propionyl‐CoA synthetase and plays a role in cancer cell growth.^16^, ^17^, ^18^

Autophagy is a cytosolic degradation and recycling process in which cells capture their own cytoplasm and organelles and consume them in lysosomes.^19^, ^20^ Recent studies have shown the importance of autophagy in regulating energy metabolism, where dysregulation of autophagy contributes to the development of metabolic disorders.^21^ The role of autophagy has also been investigated in adipocytes, and hyperactive autophagy has been observed in the WAT of obese and diabetic patients and mice.^22^, ^23^ Deletion of autophagy‐related gene 7 (*Atg7*) in adipocytes results in decreased WAT mass, increased BAT mass and enhanced insulin sensitivity.^24^, ^25^ Similarly, deletion of *Atg5* or *Atg12* also helps maintain thermogenic capacity and protects mice against diet‐induced obesity and insulin resistance.^26^ As such, pharmacological inhibition of autophagy in adipocytes using small molecule inhibitors, for example chloroquine (CQ) and hydroxychloroquine (HCQ), represents a promising approach to prevent and treat various metabolic diseases.^26^, ^27^, ^28^, ^29^ However, what triggers autophagy in adipocytes under various disease conditions are unclear.

In this study, we show that ACSS3 is a mitochondrial inner membrane protein highly enriched in BAT and is required for propionate metabolism. Using a novel Acss3^–/–^ mouse model, we further show that deletion of *Acss3* reduces BAT mass and increases WAT mass, elevates autophagy and promotes lipid accumulation in adipocytes, leading to insulin resistance and systemic metabolic syndrome. Mechanistically, loss of *Acss3* causes accumulation of its substrate, propionate in serum, which subsequently induces autophagy in adipocytes. Pharmacological inhibition of autophagy by HCQ ameliorates obesity, hyperlipidaemia, hepatic steatosis and insulin resistance phenotypes in Acss3^–/–^ mice. Our study for the first time reveals the physiological function of ACSS3 in vivo and identifies an essential role of Acss3 in propionate metabolism, adipocytes autophagy, obese and insulin resistance.

## 2 MATERIALS AND METHODS

### 2.1 Mice and animal care

The *Acss3* global knockout mice were generated by Nanjing Biomedical Research Institute of Nanjing University (NBRI) in a C57BL/6J background. Two specific gRNAs were designed in the upstream of intron 1–2 and intron 2–3, which directed Cas9 endonuclease cleavage of *Acss3* to disrupt *Acss3* expression in mice. The genotypes of experimental KO and associated control animals were as follows: Acss3^–/–^ (*Acss3* homozygous knockout mice) and HET (*Acss3* heterozygous knockout mice) and WT. Mice were housed in the animal facility with free access to water and standard rodent chow food or HFD (TD.06414 Harlan). Mouse maintenance and experimental use were performed according to protocols approved by the Purdue Animal Care and Use Committee.

### 2.2 Voluntary wheel running and food intake

Two‐month‐old male WT and Acss3^–/–^ mice weighing ∼24 g were randomly housed individually in cages with a 10.16 cm diameter polycarbonate running wheel mounted to the side of the cage (Columbus Instruments, Columbus, OH, USA) for voluntary running (*N* = 8 pairs). Running wheel counts were saved to a data file every hour by an automated computer monitoring system (Columbus Instruments). The daily running distance was calculated as average from 3 days. Mice were freely accessible to water and food, while food intake were monitored daily, and shown as average food intake/mouse/day from a week.

### 2.3 Cell culture

Primary BAT and WAT SVF preadipocytes were isolated using collagenase digestion and followed by density separation. Briefly, inguinal white (iWAT) and brown adipose tissue (BAT) were minced in a 1.7 ml tube before digested in 1.25 mg/ml collagenase type I at 37°C for 60 and 45 min, respectively. Digestion was then terminated with DMEM containing 10% FBS, and filtered through 70‐mm filters to remove undigested tissues. Cells were then centrifuged at 1700 rpm for 5 min to separate the SVF preadipocytes in the pellet and lipid‐containing adipocytes in the floating layer. The freshly isolated SVF cells were seeded and cultured in growth medium containing DMEM, 20% FBS, 1% penicillin/streptomycin (P/S) at 37°C with 5% CO_2_ until reaching a ∼80% confluency, followed by replacing fresh medium every 2 days. The 3T3‐L1 (ATCC), mouse BAT (a gift from Dr. Yongxu Wang, University of Massachusetts Medical School) and human A41 white preadipocyte cell lines (ATCC) were cultured in DMEM with 10% FBS in the same incubator. For adipogenic differentiation, mouse cells were induced with induction medium (IM, DMEM, 10% FBS, 2.85 mM insulin, 0.3 mM dexamethasone, 1 mM rosiglitazone and 0.63 mM 3‐isobutyl‐methylxanthine) for 4 days and then differentiated in differentiation medium (DM, DMEM, 10% FBS, 200 nM insulin and 10 nM T3). To induce adipogenic differentiation of human A41 cells, the culture medium was replaced with the IM containing 33 μM biotin, 0.5 μM human insulin, 17 μM pantothenate, 0.1 μM dexamethasone, 2 μM triiodothyronine, 500 μM IBMX (3‐isobutyl‐1‐methylxanthine), 30 μM indomethacin and 2% FBS and changed with fresh medium every 2 days for 11 days. Then differentiated in DM contains DMEM, 2% FBS, 200 nM insulin and 10 nM T3 (triiodothyronine) for another 4 days. To avoid the effect of cell density on adipogenic differentiation, cells were induced to differentiate when they reach 100% confluence.

### 2.4 Haematoxylin–eosin and immunofluorescence staining

Adipose tissues from the WT and Acss3^–/–^ mice were fixed in 4% PFA for 24 h at room temperature. Then the tissues were embedded into paraffin, blocked and cut at 6 mm. For H&E staining, the sections were deparaffinised, rehydrated and the nuclei stained with haematoxylin for 15 min. Sections were then rinsed in running tap water for 3 min before stained with eosin for 3 min, then were dehydrated and mounted. Images were captured using a Leica DM 6000B fluorescent microscope.

Immunofluorescence was performed in cultured SVF preadipocytes, BAT, human A41 cell lines and BAT sections. Briefly, samples were fixed in 4% paraformaldehyde for 5 min and then permeabilised and blocked in blocking buffer (PBS containing 5% goat serum, 2% bovine serum albumin, BSA, 0.2% Triton X‐100 and 0.1% sodium azide) for 1 h. Samples were subsequently incubated with primary antibodies (ACSS3, Invitrogen: PA561631, LC3B, Invitrogen: PA532254, P62, Santa Cruz: sc‐25575 and LAMP2, Abcam: ab13524) diluted in blocking buffer overnight at 4°C. After washing with PBST, the samples were incubated with secondary antibodies and DAPI for 1 h at room temperature. After final wash and mounting, fluorescent images were captured with a CoolSnap HQ charge coupled‐device camera (Photometrics) by using a Leica DM6000 microscope.

### 2.5 Blood glucose measurements

For GTT, mice were given i.p. injection of 100 mg/ml D‐glucose (2 g/kg body weight on mice with chow diet, 0.5 g/kg body weight on HFD) after overnight fasting for 14 h, and tail blood glucose concentrations were measured by a glucometer (Accu‐Check Active, Roche) every 15 min after injection continuously for 2 h. For ITT, mice were fasted for 5 h before i.p. administration of human insulin (Santa Cruz) (0.75 U/kg body weight), and tail blood glucose concentrations were monitored similarly. For both GTT and ITT, mice were caged with blinded cage number in random orders.

### 2.6 Indirect calorimetry and body composition measurement

Oxygen consumption (VO_2_) and carbon dioxide production (VCO_2_) of WT and Acss3^–/–^ mice were measured by using an indirect calorimetry system (Oxymax, Columbus Instruments). The system was kept in a stable environmental temperature (22°C) that also had a 12‐h light (6 am to 6 pm), 12‐h dark cycle (6 pm to 6 am). Mice were individually placed in each chamber with free access to food and water. Mice were adapted to the chamber for 24 h before the measurements. The data were presented as uncorrected energy expenditure levels, as well as were normalised by body weight, muscle mass and fat mass. Average energy expenditure of day (6 am to 6 pm) and night (6 pm to 6 am) values were the mean value of all points measured during the 12 h period.

Total body fat and lean mass in live animals without anaesthesia were measured by using an EchoMRI™ system located in Purdue Lily Small Animal Facility. Animals were placed in a specially sized, clear plastic holder without sedation or anaesthesia. The holder was then inserted into a tubular space in the side of the EchoMRI™ system. The animals were free to move in the holder. Each scan took about 3 min.

### 2.7 Total RNA extraction and real‐time PCR

Total RNA was extracted from cells or tissues by using Trizol Reagent according to the manufacturer's instructions. The purity and concentration of the extracted RNA were measured by a spectrophotometer (Nanodrop 3000, Thermo Fisher) at 260 and 280 nm. Ratios of absorption (260/280 nm) of all samples were made sure to be ∼2.0. Three micrograms of RNA were reversed transcribed using random primers and M‐MLV reverse transcriptase to make cDNA. Real‐time PCR was carried out with a Roche Lightcycler 480 PCR System using SYBR Green Master Mix and gene‐specific primers. Primer sequences are listed in Table S1. The 2^−ΔΔCT^ method was used to analyse the relative changes in gene expression normalised against mouse *β‐Actin* as internal control.

### 2.8 Protein extraction and western blot analysis

Protein was extracted from homogenised tissues or cells with RIPA buffer (150 mM NaCl, 1% NP‐40, 0.5% sodium deoxycholate, 0.1% SDS, 50 mM Tris‐HCl, pH 8.0) that contained a protease inhibitor cocktail (Sigma) and phosphatase inhibitors NaF and Na_3_VO_4_. Protein concentrations were determined using Pierce BCA Protein Assay Reagent (Pierce Biotechnology). Equal amount of protein from each sample was loaded for electrophoresis (Bio‐Rad). Proteins were separated by SDS–PAGE, transferred to a polyvinylidene fluoride membrane (Millipore Corporation), incubated in blocking buffer (5% fat‐free milk in TBS) for 1 h at room temperature (RT), then incubated with primary antibodies in blocking buffer overnight at 4°C. The ACSS3 (PA56163, 1:1000) and LC3B (PA532254, 1:1000) antibodies were from Invitrogen, β‐Tubulin (ab6046, 1:5000), UCP1 (ab23841, 1:1000) and mitochondrial oxidative complex cocktail (ab110413, 1: 2000) were from Abcam, other antibodies were from Santa Cruz Biotechnology (Santa Cruz), including PPARγ (sc‐7273, 1:1000), PGC1α (sc‐13067, 1:500), FABP4 (sc‐271529, 1:1000), C/EBPα (sc‐611:1000), P62 (sc‐25575, 1:200) and GAPDH (sc‐32233, 1:1000). The horseradish peroxidase (HRP)‐conjugated secondary antibody (anti‐rabbit IgG, 111‐035‐003 or anti‐mouse IgG; 115‐035‐003, Jackson ImmunoResearch) were diluted 1:20 000 and incubated at RT for 1 h. Immunodetection was performed using enhanced chemiluminescence western blotting substrate (Santa Cruz) and detected using a FluorChem R System (ProteinSimple). Results shown in the figure were representative results from at least three independent experiments.

### 2.9 Fatty acid oxidation

Fatty acid oxidation (FAO) was assessed by quantifying the production of ^14^CO_2_ from [1‐^14^C] palmitic acid. Briefly, BAT isolated from WT and Acss3^–/–^ mice was homogenised and incubated in serum‐free DMEM containing 1 mmol/L palmitic acid (500 000 DPM/ml) in 3% BSA for 3 h at 37°C and 5% CO_2_. After 3 h, the reaction was terminated by the addition of 5N perchloric acid to the cell culture dish. To trap ^14^CO_2_, 2‐phenethylamine was added to well inserts containing Whatman 1 filter papers inside of the jar. After 1 h, filter papers were placed in vials with scintillation fluid and assessed for radioactivity in a liquid scintillation counter (Tri‐Carb 1600 TR Liquid Scintillation Analyzer; PerkinElmer, Waltham, MA, USA). Oxidation values were normalised to the total protein amount of the lysate.

### 2.10 Acute cold challenge and chronic cold treatment

For chronic cold challenge, WT and Acss3^–/–^ mice were singly housed in cages and exposed to cold temperature at 4°C in an environmental chamber without food for 6 h. Rectal temperatures were measured by a digital thermometer with a probe at 0, 1, 2, 3, 4, 5 and 6 h post‐challenge. Mice were then moved out of the chamber and kept at room temperature for 7 days before cold‐exposure experiment.

For cold‐exposure experiment, WT and Acss3^–/–^ mice were singly caged and exposed to cold temperature at 6°C in an environmental chamber for 7 days as previously described.^30^ Littermate controls of WT and Acss3^–/–^ mice were maintained at room temperature in the same room as control.

### 2.11 HPLC

BAT (∼100 mg) samples were homogenised in 900 μl of water with 1.4 mm ceramic beads using a Precellys 24 homogeniser. Serum (100 μl) samples were then mixed with 900 μl water and 1.4 mm ceramic beads. The homogenates were labelled with regular aniline (^12^C_6_), and external SCFA standard solution (10 mg/ml of C2, C3 and C4) was labelled with aniline‐^13^C^6^ using N‐(3‐dimethylaminopropyl)‐N′‐ethylcarbodiimide hydrochloride (2 mg per sample). Crotonic acid (final concentration 0.1 mg/ml) was used as an internal standard. The labelling mixture was incubated for 2 h, and triethylamine was added to stop the labelling reaction. Samples and standard reaction solution were mixed (1:1) and were separated on an Agilent CN phase HPLC column and detected using Agilent 6460 triple quadrupole mass spectrometer in MRM mode. Data were collected in positive electrospray ionisation modes. SCFA concentrations in samples were determined based on peak areas of the internal standard and external standards.^31^ For statistical analysis, we used Pierce's Criterion to remove 1 data points from each propionate and butyric acid butyrate concentration results of Acss3^–/–^ BAT, that was too high and considered as ‘outliers’.

### 2.12 Short‐chain fatty acids treatment

Cultured 3T3‐L1 cells, BAT cells, human A41 cell lines and SVF preadipocytes from iWAT and eWAT were seeded onto 6‐well plates and allowed to grow until they reached 100% confluence before adding induction medium (IM). Then the cells were induced differentiation with DM as described above. Note that, 3 mM sodium acetate (C2), sodium propionate (C3) and sodium butyrate (C4) were added with DM to treat the cells for 4 days. Cells treated with 0.4 μg/ml rapamycin was set as positive control. HCQ (0.1 μg/ml) or wortmannin (0.1 mM) was used for inhibition of autophagy. RNA and protein were collected as described above for real‐time PCR and western blot analysis. Cells fixed with 4% paraformaldehyde were used for immunofluorescence as described above.

### 2.13 Rapamycin injection

Rapamycin (Calbiochem) was administered by i.p. injection at the dose of 4 mg/kg body weight per day for continuously 7 days. For preparation, rapamycin was first dissolved in ethanol at 10 mg/ml and diluted in 5% Tween‐80 (Sigma) and 5% PEG‐400 (Hampton Research) before injection.^32^

### 2.14 HCQ treatment

HCQ (H1306, TCI) was dissolved in drinking water at a concentration of 0.15 mmol/L (∼62.5 mg/L), in order to achieve an oral dosage of 10 mg/kg body weight/day of HCQ.^33^ The calculation of the HCQ dosage was based on that the average consumption of liquid was 4 ml/mouse per day for adult 2.5‐month‐old mouse with body weight around 25 g. Drinking water was adjusted to pH 7.4 with sodium hydroxide and filtrated with 0.22 μm filters to sterile. Fresh water was provided thrice weekly. WT and Acss3^–/–^ mice were administrated HCQ uninterruptedly through drinking water for 10 weeks together with or without high‐fat diet. Littermates administrated normal drinking water were set as control animals.

### 2.15 Statistical analysis

Trial experiments or experiments done previously were used to determine sample size with adequate statistical power. The researchers involved in the in vivo treatments were not completely blinded, but all ITT and GTT were conducted blindly. All images in the study were randomly captured from samples and analysed in a blinded manner. No data were excluded from statistical analysis unless mentioned. All experimental data were represented as mean ± SEM (*n* ≥ 3) and statistical comparisons were based on Student's *t*‐test with a two‐tail distribution. Comparisons with *p* values <.05 or <.01 were considered statistically significant. All presented graphs were generated by GraphPad prism software.

## 3 RESULT

### 3.1 *Acss3* is highly expressed in BAT, upregulated during adipogenic differentiation and cold‐induced thermogenesis

**FIGURE 1:**
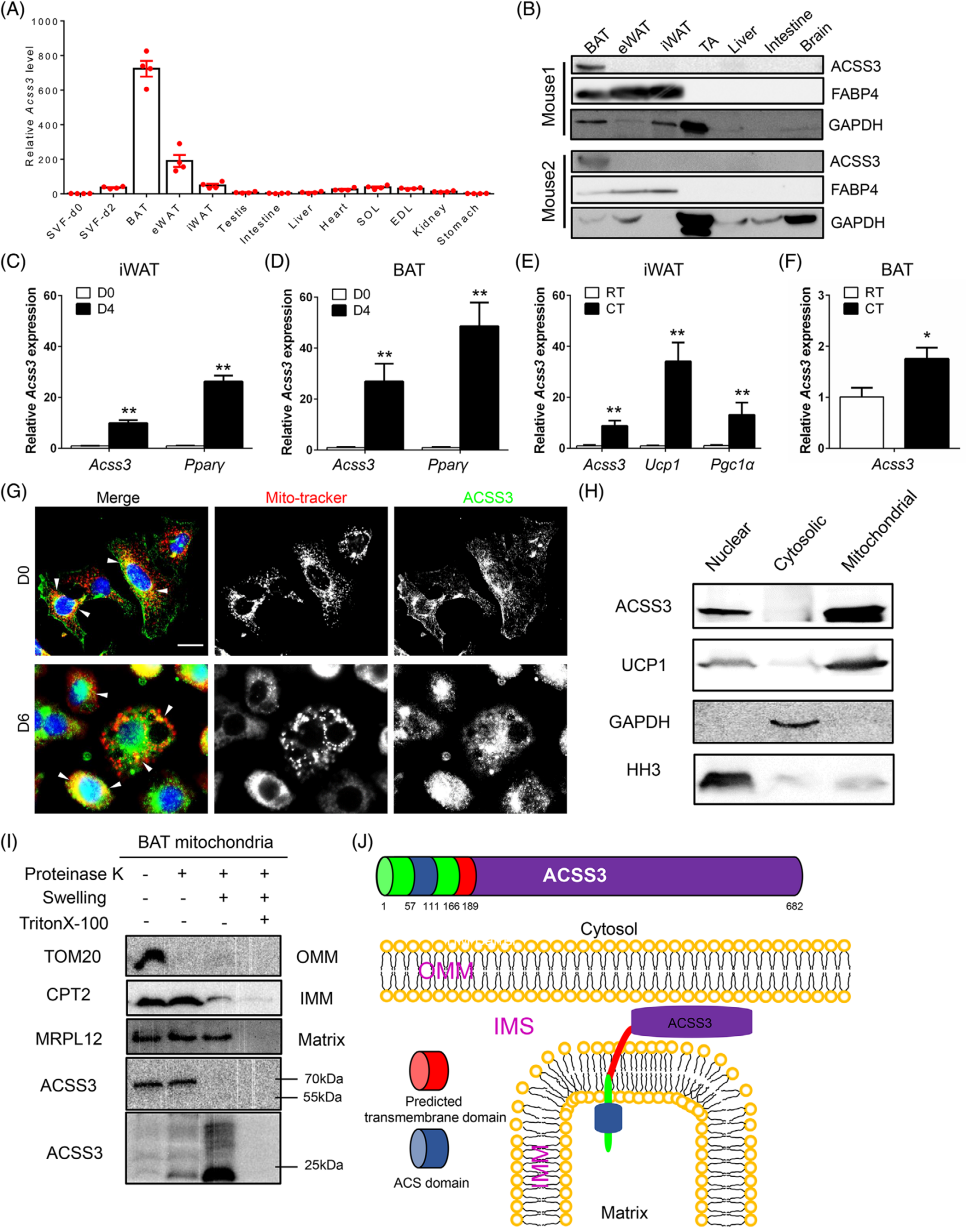
ACSS3 is a mitochondrial inner membrane protein highly expressed in brown adipose tissue. (A) qRT‐PCR detection of *Acss3* expression in different mouse tissues (*n* = 4). (B) Western blot analysis of the ACSS3 protein level in different mouse tissues (*n* = 2). (C, D) Relative levels of *Acss3* and *Pparγ* after adipogenic differentiation of preadipocytes isolated from mouse iWAT (C) and BAT (D) (*n* = 4). (E) Relative levels of *Acss3*, *Ucp1* and *Pgc1α* from iWAT after 7 days of cold treatment (*n* = 3). (F) Relative level of *Acss3* in BAT after 7 days of cold treatment (*n* = 3). (G) Co‐staining of ACSS3 and Mito‐tracker in undifferentiated (D0) and differentiated (D6) BAT cells in vitro. White arrowheads indicate the co‐localisation. Scale bar: 20 μm. (H) Western blot analysis of cell fractionation from mouse BAT (nuclear protein: Histone H3; cytosolic protein: GAPDH; mitochondrial protein: UCP1). (I) Immunoblot analysis of isolated BAT mitochondria after proteinase K (PK) accessibility test. Mitochondria were left untreated, swollen to rupture of the outer membrane, or lysed in Triton X‐100 prior to treatment with proteinase K. MRPL12 is a mitochondrial matrix protein, CPT2 is an inner membrane (IMM) protein and TOM20 is a mitochondrial outer membrane (OMM) protein. (J) A model indicates the mitochondrial localisation of ACSS3 protein. Data represent mean ± SEM (*t*‐test: *p < .05, **p < .01)

We first surveyed the expression of Acss3 in various tissues. The highest mRNA level of *Acss3* was found in BAT, followed by much lower levels in epididymal WAT (eWAT), iWAT, SVF cells and other tissues (Figure 1A). It was surprising that *Acss3* mRNA levels were very low in several metabolic tissues, including skeletal muscle, heart, liver, intestine and kidney (Figure 1A). Consistently, the protein levels of ACSS3 were only detectable in BAT and could be barely detected from other tissues (Figure 1B). We also surveyed the mRNA levels of *Acss3* during differentiation of SVF cells isolated from iWAT and BAT. Compared with day 0 (proliferating SVF cells), *Acss3* and *Pparg* levels were robustly upregulated after 4 days of adipogenic differentiation, with much higher fold‐change in BAT cells than in iWAT cells (Figure 1C, D). We further examined *Acss3* expression from ATs in response to cold‐induced thermogenesis, which upregulated *Ucp1* and *Pgc1a* in iWAT (Figure 1E). After 7 days of cold treatment (CT), the mRNA levels of *Acss3* were upregulated in both iWAT and BAT, compared to the levels of room temperature (RT) controls (Figure 1E, F).

To further pinpoint the cell population(s) that express *Acss3* in BAT and WAT, we retrieved and analysed single‐cell RNA sequencing (scRNA‐seq) data on BAT and eWAT of control and CL‐316243 (CL)‐treated mice.^34^, ^35^ Unsupervised clustering of 3602 primary cells isolated from BAT of 10‐week‐old male WT mice identified 6 clusters of cells (Figure S1A) that expressed different levels of *Ucp1* (Figure S1B).^34^ Interestingly, *Acss3^+^* cells were mainly found in Cluster 0 which was the largest *Ucp1*‐High subset and to a lesser extent in Cluster 4 (Figure S1C). Unsupervised clustering of Lin^−^ cells from eWAT of control and adrenergic agonist CL‐treated mice identified seven major clusters (Figure S2A). *Acss3^+^* cells were mainly found in Clusters 0 and 1 (*Pdgfrα^+^* progenitor cells), and the CL‐treatment did not alter the abundance of Acss3^+^ cells (Figure S2B, C). Interestingly, a subset of Acss3^+^ cells appeared in Cluster 4 in response to CL‐induced browning and *Ucp1* expression (Figure S2B, C). UAMP embedding of Cluster 4 cells from CL treatment group revealed a beige adipocyte adipogenic trajectory through expression patterns of *Adipoq, Pdgfrα* and *Ucp1* (Figure S2D, E). Consistently, expression of *Acss3* mirrors *Ucp1* expression in response to CL treatment (Figure S2D, E). Collectively, these lines of evidence demonstrate that *Acss3* is highly expressed in Ucp1^+^ brown adipocytes, and the expression is upregulated during adipogenesis and thermogenesis.

As *Acss3* expression was associated with the differentiation and thermogenesis of adipocytes, we next sought to identify potential upstream regulators of *Acss3*. To achieve this, we first analysed the transcription factor binding sites in the promoter region of *Acss3* gene. This analysis led to the identification of two conserved C/EBPα and one PPARγ binding sites within 2 Kb upstream of *Acss3* translational start site (Figure S3A), conserved in the human *ACSS3* locus.^17^ To test whether CEBPα or PPARγ transcriptionally regulated the expression of *Acss3*, we subcloned the 2 kb promoter DNA of *Acss3* into the Pgl3 plasmid (Pgl3‐*Acss3*‐P) and then performed dual luciferase reporter assay. The relative luciferase activity of cells co‐transfected with C/EBPα and Pgl3‐*Acss3*‐P was about 14‐fold higher than cells transfected with Pgl3‐*Acss3*‐P plasmid alone (Figure S3B). In contrast, cells co‐transfected with PPARγ and Pgl3‐*Acss3*‐P showed no increment of luciferase activity. These results provide compelling evidence that C/EBPα is an upstream transcriptional regulator of *Acss3* transcription.

### 3.2 ACSS3 is mainly localised to the inner membrane of the mitochondria

To investigate the subcellular localisations of ACSS3 in BAs, we co‐stained ACSS3 and Mito‐Tracker. The staining indicated an obvious co‐localisation of ACSS3 with mitochondria in undifferentiated BAT cells (Figure 1G). After 6 days of differentiation, both mitochondria and ACSS3 signals were increased and largely overlapped (Figure 1G). We then performed cell fractionation to separate mitochondrial, cytosolic and nuclear proteins from BAT of 2‐month‐old WT mice. ACSS3 were detected in the mitochondrial and nuclear fractions together with mitochondrial inner membrane protein UCP1, but not detectable in the cytosolic fraction that expresses GAPDH (Figure 1H). The expression of mitochondrial proteins in the nuclear fraction suggests that a significant subset of mitochondria is closely associated with the nuclear outer membrane in BAs. We subsequentially determined the submitochondrial localisation of ACSS3 using proteinase K (PK) accessibility test (Figure 1I). Mitochondria isolated from BAT of 2‐month‐old mice were left untreated, swollen to rupture of the outer membrane, or lysed in Triton X‐100 prior to treatment with PK to reveal outer membrane protein, inner membrane protein and matrix protein, using TOM20, CPT2 and MRPL12 as reference proteins, respectively (Figure 1I). We found that ACSS3 was not protected from Proteinase K digestion after swelling, indicating its inner membrane localisation (Figure 1I). A truncated ACSS3 band (∼20 kDa) recognised by the antibody raised against an N‐terminus epitope was protected from PK digestion after swelling, suggesting that the N‐terminal domain of ACSS3 was in the matrix (Figure 1I). Using an online tool called TMHMM (prediction of transmembrane helices in proteins), we also predicted a transmembrane domain of 24 AAs (from 166–189) in the ACSS3 protein (Figure 1J). Based on the results, we hypothesised that the ACS domain (AA 57–111) should localise in the mitochondrial matrix (Figure 1J). Supporting this, the predicted molecular size of the N‐terminal 189 AAs of ACSS3 containing the ACS domain and transmembrane domain was ∼20.5 kDa, consistent with the observed size that was protected from Proteinase K digestion after swelling (Figure 1J).

### 3.3 Loss of *Acss3* leads to reduced BAT but increased WAT mass in mice

**FIGURE 2:**
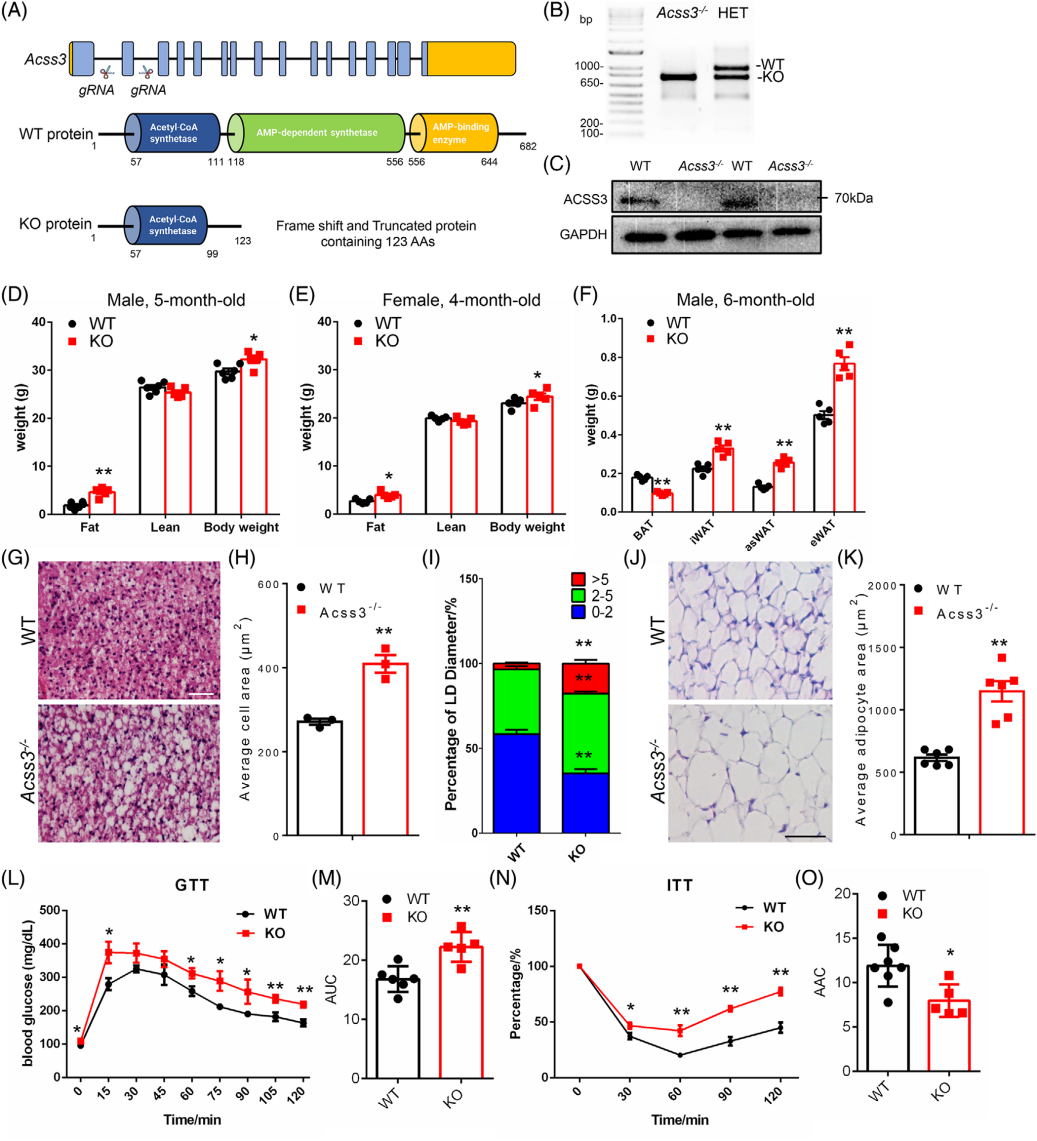
*Acss3* KO reduces BAT mass and increases WAT mass, impairing systemic metabolism. (A) Targeting strategy for global *Acss3* knockout mice (Acss3^–/–^) by using CRISPR‐Cas9 gene editing. Upper: *Acss3* gene structure showing exons (blue boxes) and 5′ and 3′ untranslated regions (yellow boxes), scissors indicates the target site of two gRNAs. Middle: ACSS3 protein domain structure with amino acids numbers labelled. Acetyl‐CoA synthetase (ACS) is the catalytic domain. Lower: excision of exon2 results in reading frame shift and a premature translational stop codon, generating a truncated protein containing only partial of the ACS domain. (B) Successful deletion of exon2 is shown by DNA recombination using ear DNA from Acss3^–/–^ and heterozygous (HET) knockout mice. (C) Completely deletion of ACSS3 protein level in brown adipose tissue (BAT) of 6‐month‐old male Acss3^–/–^ mice. (D, E) Compared to WT mice, Acss3^–/–^ leads to increased total body fat and body weight. *N* = 6 and 5 pairs of male (D) and female (E) mice for 5‐ and 4‐month‐old, respectively. (F) Weights of various BAT and WAT [epididymal white adipose tissue (eWAT); inguinal white adipose tissue (iWAT) and anterior subcutaneous white adipose tissue (asWAT)] depots from male mice at 6‐month‐old, *N* = 5 pairs. (G) H&E staining of BAT from WT and Acss3^–/–^ mice at 6‐month‐old. Scale bar: 50 μm. (H) Average cell areas of WT and Acss3^–/–^ BAT, *N* = 3 pairs, calculated by counting the total nuclei numbers per image, 3 images per mice. (I) Percentage of LD diameters of BAT from WT and Acss3^–/–^ mice. (J) H&E staining of eWAT from WT and Acss3^–/–^ mice at 6‐month‐old. Scale bar: 50 μm. (K) Average adipocyte areas of WT and Acss3^–/–^ eWAT, *N* = 6, calculated by averaging 100 adipocytes per image, 3 images per mice. (L, M) Blood glucose concentrations during glucose tolerance test (GTT) performed on 3‐month‐old Acss3^–/–^ mice (L). Area under curve (AUC) calculated based on data in M. *N* = 6 and 5 mice for WT and Acss3^–/–^, respectively. (N, O) Percentage changes of blood glucose concentrations during insulin tolerance test (ITT) performed on 3‐month‐old Acss3^–/–^ mice (N). Area above curve (AAC) calculated based on data in O. *N* = 7 and 5 mice for WT and Acss3^–/–^, respectively. Data represent mean ± SEM (*t*‐test: *p < .05, **p < .01)

**FIGURE 3:**
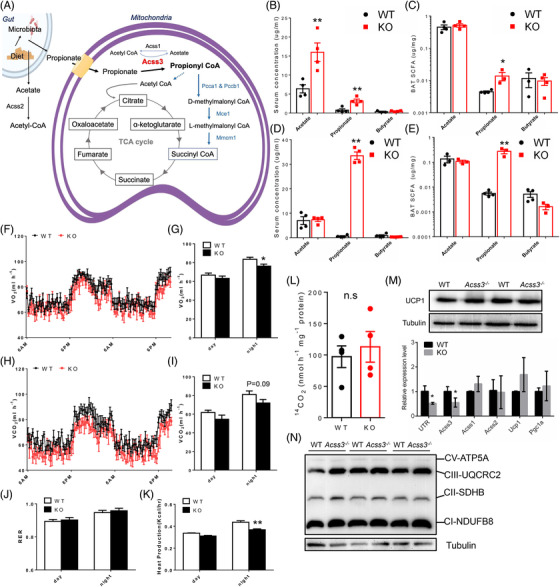
*Acss3* deletion causes propionate accumulation and impairs energy expenditure in mice. (A) A diagram showing production of propionate in gut and the role of ACSS3 in Propionyl‐CoA biosynthesis and TCA cycle within mitochondrial matrix. (B, C) Concentrations of acetic acid (acetate), propionic acid (propionate) and butyric acid (butyrate) from serum (B) and BAT (C) of 2‐month‐old Acss3^–/–^ and WT mice. *N* = 4 pairs. (D, E) Concentrations of acetate, propionate and butyrate from serum (D) and BAT (E) of Acss3^–/–^ and WT mice after 10 weeks of HFD feeding. *N* = 4 pairs. (F–I) O_2_ consumption (F) measured by an indirect calorimetry is shown for a 36‐h cycle, average day and night O_2_ consumption (VO_2_, G), 36‐h cycle of CO_2_ production (H), average day and night CO_2_ production (VCO_2_, I) of 3‐month‐old WT and Acss3^–/–^ mice. *N* = 5 pairs of mice. (J, K) Average day and night respiratory exchange ratio (RER, J) and heat production (K) of 3‐month‐old WT and Acss3^–/–^ mice. *N* = 5 pairs of mice. (L) Fatty acid oxidation measured by ^14^CO_2_ production of BAT lysate from WT and Acss3^–/–^ mice incubated with [1‐^14^C] palmitic acid. (M) Western blot analysis of UCP1 in BAT lysate (upper), and relative levels of *Acss3*, *Acss1*, *Acss2*, *Ucp1* and *Pgc1α* (lower, *N* = 4), from WT and Acss3^–/–^ mice. UTR: a pair of primers detects the *3′ UTR* of *Acss3* mRNA. (N) Western blot analysis of mitochondrial OXPHOS complexes from BAT lysis. *N* = 3 pairs of WT and Acss3^–/–^ mice. Data represent mean ± SEM (*t*‐test: *p < .05, **p < .01)

To investigate the role of ACSS3 in vivo, we generated a global *Acss3* knockout mouse (Acss3^–/–^) model (Figure 2A). In Acss3^–/–^ mice, exon 2 of *Acss3* was deleted, leading to premature translational stop and generation of a truncated peptide completely lacking the key functional domains including the AMP‐dependent synthetase domain and AMP‐binding enzyme domain (Figure 2A). We confirmed the deletion of exon 2 by DNA recombination from mouse ear DNA of both heterozygous knockout mice and Acss3^–/–^ mice (Figure 2B). We also confirmed that ACSS3 protein was completely diminished in Acss3^–/–^ compared to WT BAT (Figure 2C). Acss3^–/–^ mice were born at expected Mendalian ratio, and morphologically indistinguishable from their heterozygous and WT littermates. However, as Acss3^–/–^ mice grew, they began to appear larger than WT mice. At 5‐month‐old, the body weight of male Acss3^–/–^ mice was significantly heavier than that of WT mice (Figure 2D). EchoMRI body composition scanning showed that the body weight difference was mainly due to differences in total body fat mass (Figure 2D). At 5‐month‐old, the total body fat mass of the Acss3^–/–^ mice was ∼2.5‐fold larger than that of WT mice (Figure 2D). Consistently, 4‐month‐old female Acss3^–/–^ mice also exhibited increased body weight and total body fat mass (Figure 2E). We isolated various fat depots from 6‐month‐old male Acss3^–/–^ and WT mice (Figure S4A) and confirmed that while the BAT mass was decreased, the WAT masses were increased in Acss3^–/–^ mice (Figure 3F), and the increase was more prominent in visceral eWAT (Figure 3F). H&E staining revealed that the average cell size and LD size of BAs was obviously larger in 6‐month‐old Acss3^–/–^ than age‐matched WT mice (Figure 2G–I). In addition, the average size of adipocytes from eWAT was also obviously larger in 6‐month‐old Acss3^–/–^ compared to that of age‐matched WT mice (Figure 2J, K). By contrast, there were no differences in the weights of other tissues, such as skeletal muscle, liver, heart and kidney, from 6‐month‐old male Acss3^–/–^ and WT mice (Figure S4B, C). Taken together, deletion of *Acss3* decreases BAT mass and increases WAT mass in mice.

Further inspections demonstrated that 2‐month‐old Acss3^–/–^ mice also exhibited reduced BAT mass, but there was no difference in the body weight nor WAT mass (Figure S5A, B). However, the sizes of LD and BAs from BAT of Acss3^–/–^ mice were significantly smaller than those of WT mice at 2‐month‐old (Figure S5C, D). Similar results were also observed in the mice at 6‐week‐old and postnatal day 7 (P7) (Figure S5E–N), with reduced BAT mass, smaller BA size in Acss3^–/–^ mice, but no change in WAT mass and cell size. Consistently, the protein levels of UCP1 were not changed, but the FABP4 were decreased in Acss3^–/–^ BAT compared to those of WT BAT (Figure S5O). We then cultured preadipocytes from BAT of WT and Acss3^–/–^ mice and induced the SVF cells to differentiate (IM 4d + DM 4d) into LD‐laden adipocytes. Compared to WT cells, there were less LDs in Acss3^–/–^ BA (Figure S5P). These results demonstrated that deletion of *Acss3* in adipocytes leads to reductions in BAT mass accumulation that may be due to impaired BA differentiation.

### 3.4 Loss of *Acss3* disrupts BA differentiation in vitro

In view of the Acss3 expression pattern and impaired BA differentiation, we further performed the loss‐of‐function analysis in the immortalised murine brown preadipocytes (brown adipocyte cell line) with two independent lentiviral shRNAs. The transfection of shRNA1 and shRNA2 achieved 87% and 48% reduction of *Acss3* mRNA in BAT cell line, respectively (Figure S6A). Therefore, both shRNA1 and shRNA2 lentivirus were then used to establish stable *Acss3* knockdown (KD) brown adipocyte cell lines (sh1 and sh2). The total cell count result showed that *Acss3* KD did not affect proliferation of BAT cells (Figure S6B). We then differentiated the BA cell lines and used Bodipy staining to visualise lipid droplets (LDs). The results showed that *Acss3* KD dramatically reduced the LD numbers compared to control cells after 6 days of differentiation (Figure S6C). We further investigated how *Acss3* KD affected differentiation of BA by profiling expression of genes involved in adipogenesis, lipogenesis, lipolysis and browning. The mRNA levels of *Fasn*, *Adipoq*, *Pparγ, C/ebpα* and *Fabp4* were significantly lower in sh1 and sh2 compared to control cells (Figure S6D). Expression levels of browning related genes *Ucp1*, *Prdm16* and *Pgc1α* were also decreased after *Acss3* KD (Figure S6E). Consistently, the protein levels of PLIN1, PGC1α and C/EBPα were also lower in sh1 and sh2 than control cells after differentiation (Figure S6F, G). Consistent with the impaired adipogenesis after *Acss3* KD, the mRNA levels of *Cox5b*, *Cox7a*, *Cox8b* and *Cpt2*, as well as the protein levels of mitochondrial OXPHOS complexes, including ATP5A, UQCRC2 and SDHB, were all downregulated in sh1 and sh2 compared to control cells after differentiation (Figure S6J, K). These data indicate that *Acss3* KD inhibits the adipogenesis and mitochondrial biogenesis of BA in vitro.

### 3.5 Knockout of *Acss3* causes serum propionate accumulation and impaired systemic metabolism

We next examined how loss of *Acss3* affected the systemic metabolism of mice. We first conducted glucose tolerance tests (GTT) to determine glucose disposal after intraperitoneal (i.p.) injection of glucose into Acss3^–/–^ and WT mice at 3‐month‐old (Figure 2L, M). Acss3^–/–^ mice had higher blood glucose levels than the WT littermates after injection of glucose (Figure 2L). Area under curve (AUC) of Acss3^–/–^ mice was also significantly larger than that of WT mice (Figure 2M), suggesting that Acss3^–/–^ mice were glucose intolerant. We next performed insulin tolerance tests (ITT) to determine how blood glucose level changed in response to insulin injection (Figure 2N, O). Acss3^–/–^ mice (3‐month‐old) exhibited impaired insulin sensitivity, manifested by decreased rates of insulin‐mediated glucose clearance and lower area above curve (AAC) in Acss3^–/–^ compared to WT mice (Figure 2N, O). These results together demonstrate that loss of *Acss3* disrupts glucose homeostasis and insulin sensitivity of the mice.

It has been reported that ACSS3 catalyses the formation of propionyl‐CoA through activating propionic acid, or propionate (Figure 3A); we therefore examined if *Acss3* KO impaired propionate metabolism in mice. We performed high‐performance liquid chromatography (HPLC) to quantify the concentrations of acetic acid (acetate), propionic acid (propionate) and butyric acid (butyrate) from serum and BAT of 2‐month‐old Acss3^–/–^ and WT mice. Propionic acid concentrations were increased by ∼4‐fold while acetic acid was elevated by ∼2.5‐fold in serum of 2‐month‐old Acss3^–/–^ than those of WT mice (Figure 3B). In addition, the propionic acid concentration in Acss3^–/–^ BAT was increased by ∼3‐fold compared with WT (Figure 3C), and there was no difference in the acetic acid contents of BAT at 2‐month‐old (Figure 3C). After fed with HFD for 10 weeks, serum propionic acid concentration was dramatically increased in Acss3^–/–^ mice (Figure 3D), but there was no difference in the serum acetic acid concentration after HFD (Figure 3D). Consistently, there was also a huge increase in the propionic acid concentration in BAT of Acss3^–/–^ compared to that of WT after HFD (Figure 3E). For butyric acid, it remained at low concentrations in all the tested samples and showed no difference across groups. Thus, these results show that *Acss3* deletion leads to specific accumulation of propionate in BAT and serum, suggesting that ACSS3 is indispensable for propionate metabolism.

We further checked the respiration and energy expenditure of WT and Acss3^–/–^ mice using an indirect calorimetry approach. Acss3^–/–^ mice had lower levels of oxygen (O_2_) consumption and carbon dioxide (CO_2_) production than WT mice at 3‐month‐old (Figures 3F,G and S7A–C), which were only significant at night when mice actively feed. Carbon dioxide (CO_2_) production of Acss3^–/–^ mice also showed a trend to increase, but did not reach a significant level (Figure 3H, I). Consequently, the heat production was also decreased in Acss3^–/–^ mice compared to that of WT mice at night (Figure 3J, K). The respiratory exchange ratio (RER) did not show any difference between WT and Acss3^–/–^ mice. These results together demonstrate that accumulation of propionate in Acss3^–/–^ mice disrupts the systemic metabolism. Since *Acss3* is highly expressed in BAT, we then analysed fatty acid oxidation (FAO) of WT and Acss3^–/–^ BAT after incubation with ^14^C‐labelled palmitate. However, the BAT FAO indicated by ^14^CO_2_ production was similar in BAT from Acss3^–/–^ compared to WT mice (Figure 3L). Protein level of UCP1 as well as the mRNA levels of *Ucp1* and *Pgc1α* were not changed in Acss3^–/–^ compared to those in WT BAT (Figure 3M). In addition, no compensatory expressions of *Acss1* and *Acss2* were observed (Figure 3M). Moreover, the protein levels of mitochondrial OXPHOS complexes were also identical in WT and KO BAT (Figure 3N).

To further access the function of BAT, we performed both acute cold challenge and 7‐day chronic cold treatment (CT). The rectal temperatures of Acss3^–/–^ mice were identical to those of WT mice except for a ∼0.5°C drop at 2 h after acute could challenge (Figure S8A). Chronic CT induced an obvious red appearance suggestive of increased angiogenesis, as well as significant weight loss of WAT from both Acss3^–/–^ and WT mice (Figure S8B). BAT mass was decreased while WAT masses were increased in 6‐month‐old male Acss3^–/–^ mice at both RT and CT (Figure S8C), and more prominent in eWAT than iWAT (Figure S8C). The mRNA levels of *Ucp1* and *Pgc1α* were not different in Acss3^–/–^ mice compared to WT mice at both RT and CT (Figure S8D). Expression levels of *Acss1* and *Acss2* were not changed, excluding potential compensation by these family members (Figures 3M and S8D). LD size of BA as well as the adipocytes size of iWAT were obviously larger in Acss3^–/–^ mice than age‐matched WT mice after CT (Figure S8E, F). In addition, H&E staining also showed that the iWAT from Acss3^–/–^ mice contained less clusters of pink staining than WT mice after CT. These results indicate that loss of *Acss3* has minor impact on BAT mitochondria function and Acss3^–/–^ mice had normal capacity for cold‐induced browning.

The increased serum propionate levels and associated obesity and insulin resistance of the Acss3^–/–^ mice prompted us to examine if higher serum propionate concentration was correlated to obesity and T2D in humans. We retrieved data from published studies and found that the production of propionate by gut microbiota (Figure S9A) as well as the serum propionate levels (Figure S9B) were indeed increased in obese and T2D patients.^36^, ^37^ To further establish whether *ACSS3* level is correlated to obesity and T2D, we retrieved data from published studies,^38^, ^39^ in which *ACSS3* expressions in WAT from lean and obese patients, as well as insulin sensitive and insulin‐resistant patients are available. The results showed that *ACSS3* expression in WAT was significantly lower in obese than lean subjects (Figure S9C), while *LAMP1* (An autophagic‐lysosomal marker^40^) levels were upregulated in the same samples (Figure S9D). Additionally, *ACSS3* expression level was significantly lower in WAT of insulin‐resistant patients than those from insulin sensitive humans (Figure S9E). These results establish a negative trend of *ACSS3* expression level, along with a positive trend of propionate accumulation, with obesity and T2D in humans.

### 3.6 Propionate induces autophagy of cultured adipocytes in vitro

**FIGURE 4:**
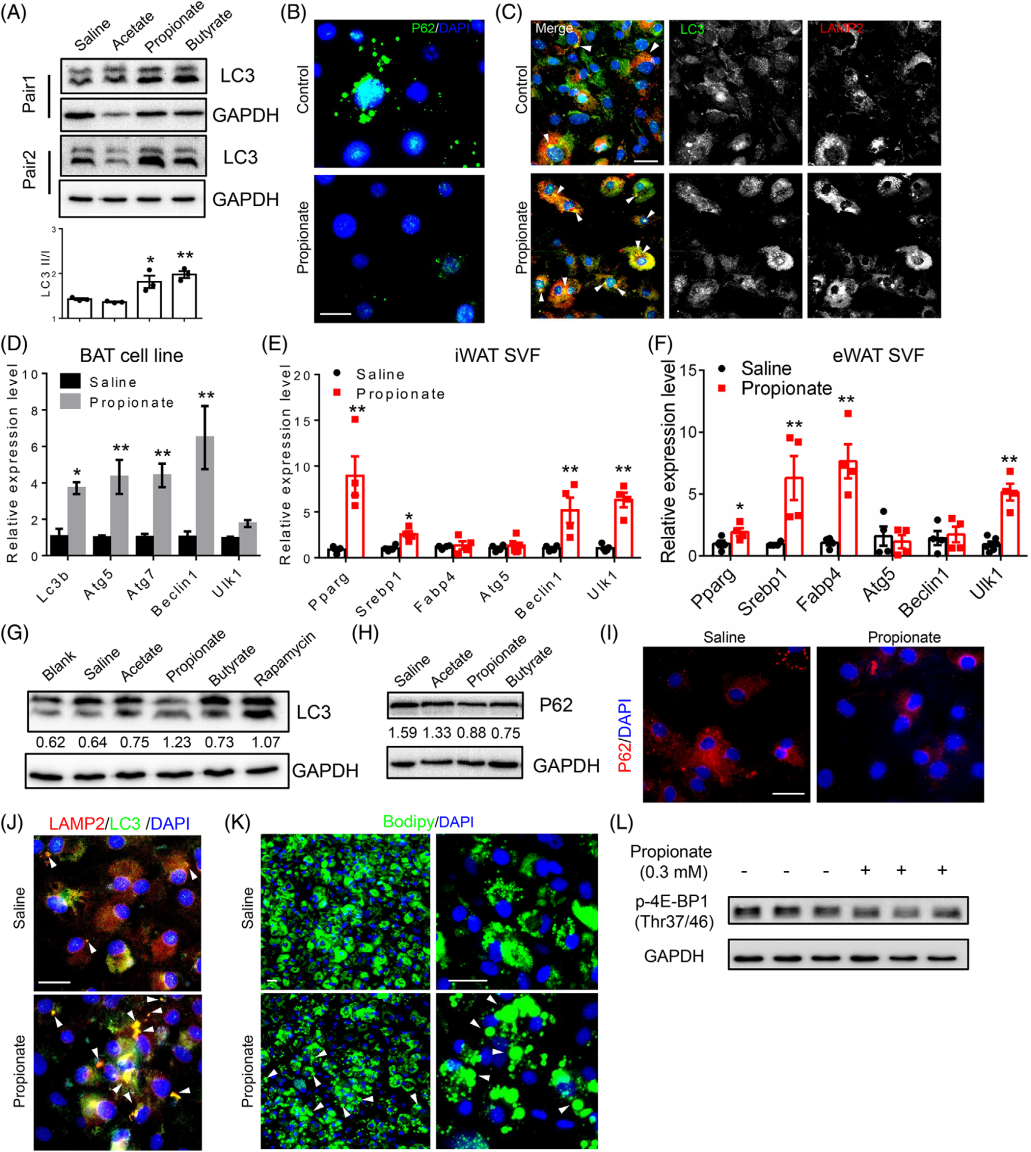
Propionate promotes autophagy of adipocytes. (A) Western blot analysis of LC3 (upper) and ratios of LC3 II/I (lower) of mouse BAT cells treated with 3 mM sodium acetate (acetate), sodium propionate (propionate) and sodium butyrate (butyrate). *N* = 3 independent treatments. (B) Staining of P62 (green) of saline‐ and propionate‐treated BAT cells. Scale bar: 10 μm. (C) Co‐staining of LC3 (green) and LAMP2 (red) in 8‐day differentiated preadipocytes isolated from BAT of WT mice treated with saline and propionate. White arrowheads indicate the co‐localisation. Scale bar: 20 μm. (D) Relative levels of *Lc3b*, *Atg5*, *Atg7*, *Ulk1* and *Beclin1* from saline‐ and propionate‐treated 6‐day differentiated BAT cell line; *N* = 4 independent treatments. (E, F) Relative levels of *Pparγ, Fabp4*, *Srebp1*, *Atg5*, *Ulk1* and *Beclin1* from saline‐ and propionate‐treated 8‐day differentiated SVF preadipocytes isolated from iWAT (E) and eWAT (F) of 8‐week‐old WT mice; *N* = 4 independent treatments. (G) Western blot analysis of LC3 of differentiated 3T3‐L1 cells treated with 3 mM sodium acetate (acetate), sodium propionate (propionate) and sodium butyrate (butyrate). Rapamycin‐treated cells were set as positive control. Numbers below the blot indicate ratios of LC3 II/I. (H) Western blot analysis of P62 from differentiated human A41 preadipocytes treated with 3 mM acetate, propionate and butyrate; numbers below the blot indicate ratio of P62/GAPDH. (I) P62 (green) staining of saline and propionate treated differentiated human A41 preadipocytes. Scale bar: 20 μm. (J) Co‐staining of LC3 (green) and LAMP2 (red) in 14‐day differentiated human A41 preadipocytes treated with saline and propionate. White arrowheads indicate the co‐localisation. Scale bar: 20 μm. (K) Bodipy staining indicates enlarged LDs of propionate‐treated human white adipocytes. White arrowheads indicate the larger LDs after propionate treatment. Scale bar: 20 μm. (L) Western blot analysis of p‐4E‐BP1 (Thr37/46) of human A41 adipocytes treated with 0.3 mM sodium propionate. *N* = 3 independent treatments. Data represent mean ± SEM (*t*‐test: *p < .05, **p < .01)

Propionate and butyrate are reported to induce autophagy in human colon cancer cells.^28^ To determine whether SCFA, especially propionate, could induce autophagy of adipocytes, we first treated differentiated brown adipocyte cell line with acetate, propionate and butyrate with a final concentration of 3 mM. We found that the protein levels of LC3 II and the ratios of LC3 II/I, markers of autophagy were significantly increased in differentiated brown adipocytes after propionate and butyrate treatment (Figure 4A). Furthermore, propionate treatment did not affect the cell viability of cultured adipocytes as indicated by crystal violet staining (Figure S10). By using a lower propionate concentration (0.3 mM), we were also able to detect an increased LC3 II/I ratio after 24 h of treatment (Figure S11A). We also performed immunostaining of P62 (a negative marker of autophagy), LC3 and LAMP2 (a lysosome marker). The results showed a clear decrease in P62 puncta number in propionate treated compared with saline‐treated brown adipocytes (Figure 4B). Co‐staining of LC3 and LAMP2 also revealed increased co‐localisation of autophagosomes with lysosomes (Figure 4C), confirming elevated autophagy induced by propionate. Propionate treatment also upregulated mRNA levels of autophagy‐related genes, *Lc3b*, *Atg5*, *Atg7* and *Beclin1* in BAT cells (Figure 4D). These results demonstrate that SCFA, especially propionate, induce autophagy in brown adipocytes.

To further examine if propionate induced autophagy in white adipocytes, we isolated SVF cells from iWAT and eWAT of WT mice and treated the differentiating cells with propionate for 4 days. After propionate treatment, mRNA levels of adipogenic, lipogenic and autophagy related genes, such as *Pparγ*, *Srebp1* and *Ulk1*, were upregulated (Figure 4E, F). We also examined the autophagy of differentiated 3T3‐L1 cells after SCFA treatment. Consistently, increased LC3 II/I ratios were detected from propionate, butyrate and rapamycin (Rapa) treated compared to those of saline‐ and acetate‐treated cells (Figure 4G). We further tested these effects on cultured human WAs. We treated differentiated (11‐day IM + 4‐day DM) human A41 WAT preadipocyte cell line (hWAT) with different SCFAs as mentioned above. While the relative levels of P62 (normalised to GAPDH) were decreased after propionate and butyrate treatment (Figure 4H). Immunostaining of P62 confirmed a clear decrease in P62 puncta in propionate treated compared to those saline‐treated hWAT (Figure 4I). Increased co‐localisation of LC3 and LAMP2 from propionate‐treated hWAT further indicated elevated autophagy (Figure 4J). Notably, Bodipy staining showed that after propionate treatment, hWAT accumulated more larger LDs (Figure 4K). As reported,^31^ propionate treatment also decreased the protein levels of p‐4E‐BP‐1, a downstream target of mTOR (Figure 4L). These data collectively indicate that SCFAs, especially propionate, induce autophagy in cultured adipocytes and inhibits mTOR signalling in vitro.

### 3.7 *Acss3* deficiency elevates autophagy of adipocytes in vivo

**FIGURE 5:**
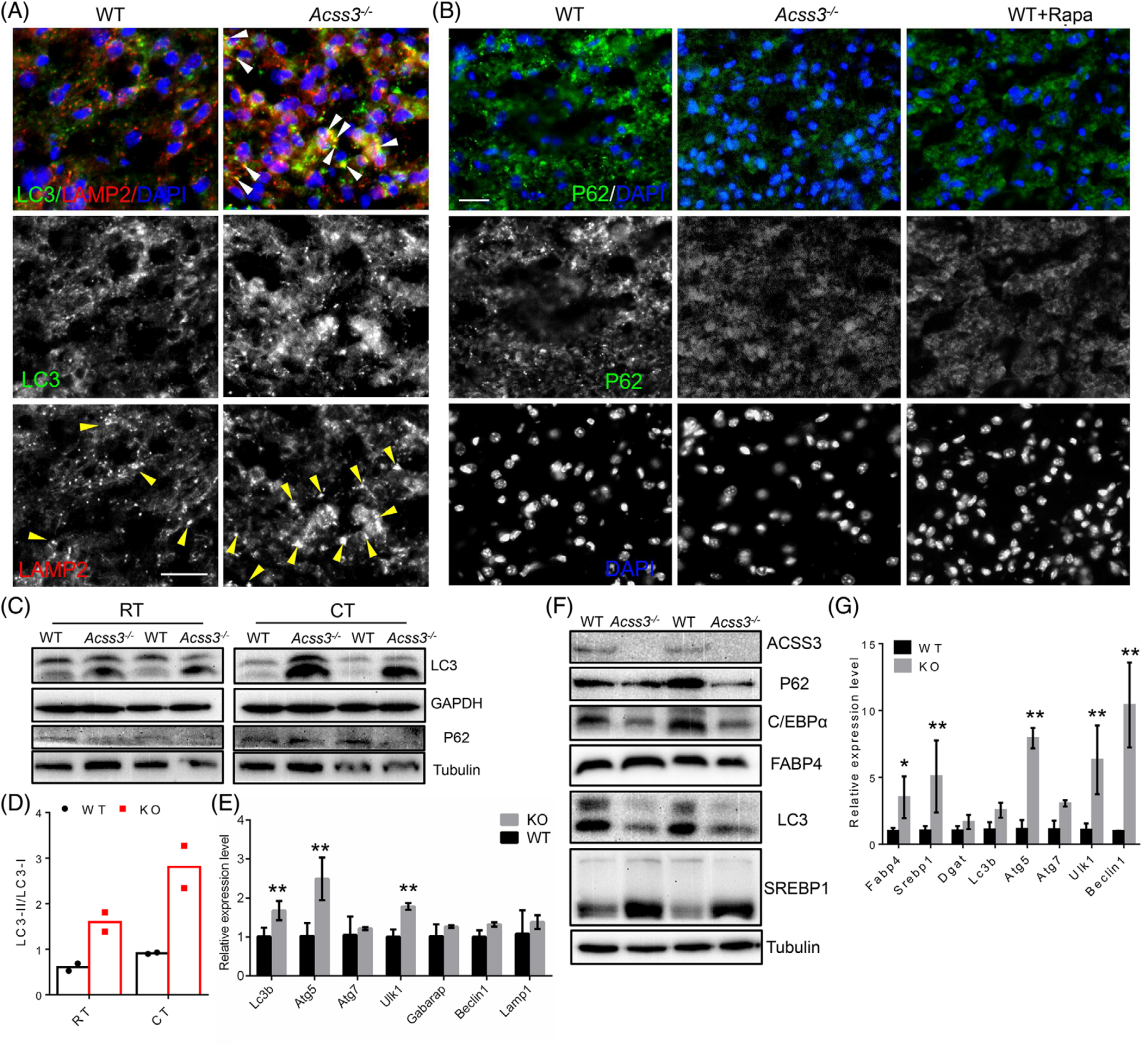
Elevated autophagy in Acss3^–/–^ adipose tissue. (A) Co‐staining of LC3 (green) and LAMP2 (red) in BAT from 3‐month‐old WT and Acss3^–/–^ mice. White arrowheads indicate the co‐localisation, and yellow arrowheads indicate large lysosomes. Scale bar: 20 μm. (B) Staining of P62 (green) in BAT from 3‐month‐old WT and Acss3^–/–^ mice, and WT mice injected with rapamycin (Rapa). Scale bar: 20 μm. (C) Western blot analysis of LC3 and P62 from BAT lysate of 6‐month‐old WT and Acss3^–/–^ mice at room temperature (RT) and after cold treatment (CT). (D) Ratios of LC3 II/I in C. (E) Relative levels of *Lc3b*, *Atg5*, *Atg7*, *Ulk1*, *Gabarap*, *Beclin1* and *Lamp1* from WT and Acss3^–/–^ mice at RT, *N* = 4 pairs of mice at 6‐month‐old. (F) Western blot analysis of ACSS3, P62, C/EBPα, FABP4, LC3 and SREBP1 from eWAT of 6‐month‐old WT and Acss3^–/–^ mice. (G) Relative levels of *Fabp4*, *Srebp1*, *Dgat*, *Lc3b*, *Atg5*, Atg7, *Ulk1* and *Beclin1* from eWAT of WT and Acss3^–/–^ mice, *N* = 3 pairs of mice at 6‐month‐old. Data represent mean ± SEM (*t*‐test: *p < .05, **p < .01)

Considering that propionate promotes autophagy of adipocytes in vitro, we next explored whether accumulation of propionate in the Acss3^–/–^ mice also promotes adipocyte autophagy in vivo. We examined BAT cross sections after immunostaining with LC3, LAMP2 and P62. We observed larger and brighter LC3 and reduced LAMP2 puncta from Acss3^–/–^ compared to WT BAT (Figure 5A). The fusion of autophagosomes with lysosomes (identified by LC3 and LAMP2 co‐localisation), were dramatically increased in Acss3^–/–^ compared to that of WT BAT (Figure 5A). Consistently, there were less P62 puncta in BAT from Acss3^–/–^ and rapamycin‐treated mice (rapamycin is an inducer of autophagy), compared with those bright and widely distributed P62 puncta in WT BAT (Figure 5B). Protein levels of LC3 II and the ratio of LC3 II/I were also obviously higher in Acss3^–/–^ than WT BAT (Figure 5C, D). Interestingly, CT exacerbated the elevation of LC3 II/I ratio and decrease of P62 levels in Acss3^–/–^ mice (Figure 5C, D). Similarly, mRNA levels of autophagy‐related genes, *Lc3b*, *Atg5* and *Ulk1* were upregulated in BAT of Acss3^–/–^ mice (Figure 5E).

Similar results were also observed in *Acss3* KO eWAT, where protein levels of P62 were downregulated in Acss3^–/–^ compared to WT eWAT (Figure 5F). Ratios of LC3 II/I were conversely increased after *Acss3* KO (Figure 5F). Interestingly, protein level of adipogenic transcription factor C/EBPα was dramatically reduced while the mature form of lipogenic transcription factor SREBP1 was significantly increased in Acss3^–/–^ eWAT (Figure 5F), indicating impaired adipogenesis but enhanced lipogenesis after *Acss3* deletion. Consistent with protein expressions, mRNA levels of *Fabp4*, *Srebp1*, *Atg5*, *Ulk1* and *Beclin1* were all upregulated from Acss3^–/–^ compared to those of WT eWAT (Figure 5G). Together, deletion of *Acss3* promotes autophagy of both brown and white adipocytes in vivo.

### 3.8 HCQ inhibition of autophagy rescues metabolic defects of Acss3^–/–^ mice

To determine if elevated autophagy is responsible for the metabolic phenotypes in the Acss3^–/–^ adipocytes and mice, we treated cells and animals with hydroxychloroquine (HCQ) and wortmannin, well‐established autophagy inhibitors. We first treated cultured WAs and BAs using 0.1 mM wortmannin, which efficiently inhibited propionate‐induced autophagy as indicated by decreased LC3 II/I ratios (Figure S11A, B). We also treated BAs and WAs with HCQ (0.1 μg/ml), which significantly restored the protein level of P62 that was decreased in propionate treated BAT cells (Figure S11C), suggesting that HCQ inhibits autophagy in BAs. Immunostaining also confirmed that propionate decreased P62 immunofluorescence, which was robustly restored by HCQ treatment (Figure S11D). In addition, in differentiated hWAT, HCQ treatment blocked propionate‐induced autophagy as indicated by restoration P62 protein levels (Figure S11E). These results collectively demonstrate that HCQ/wortmannin treatment efficiently inhibits propionate‐induced autophagy in cultured BAs and WAs.

**FIGURE 6:**
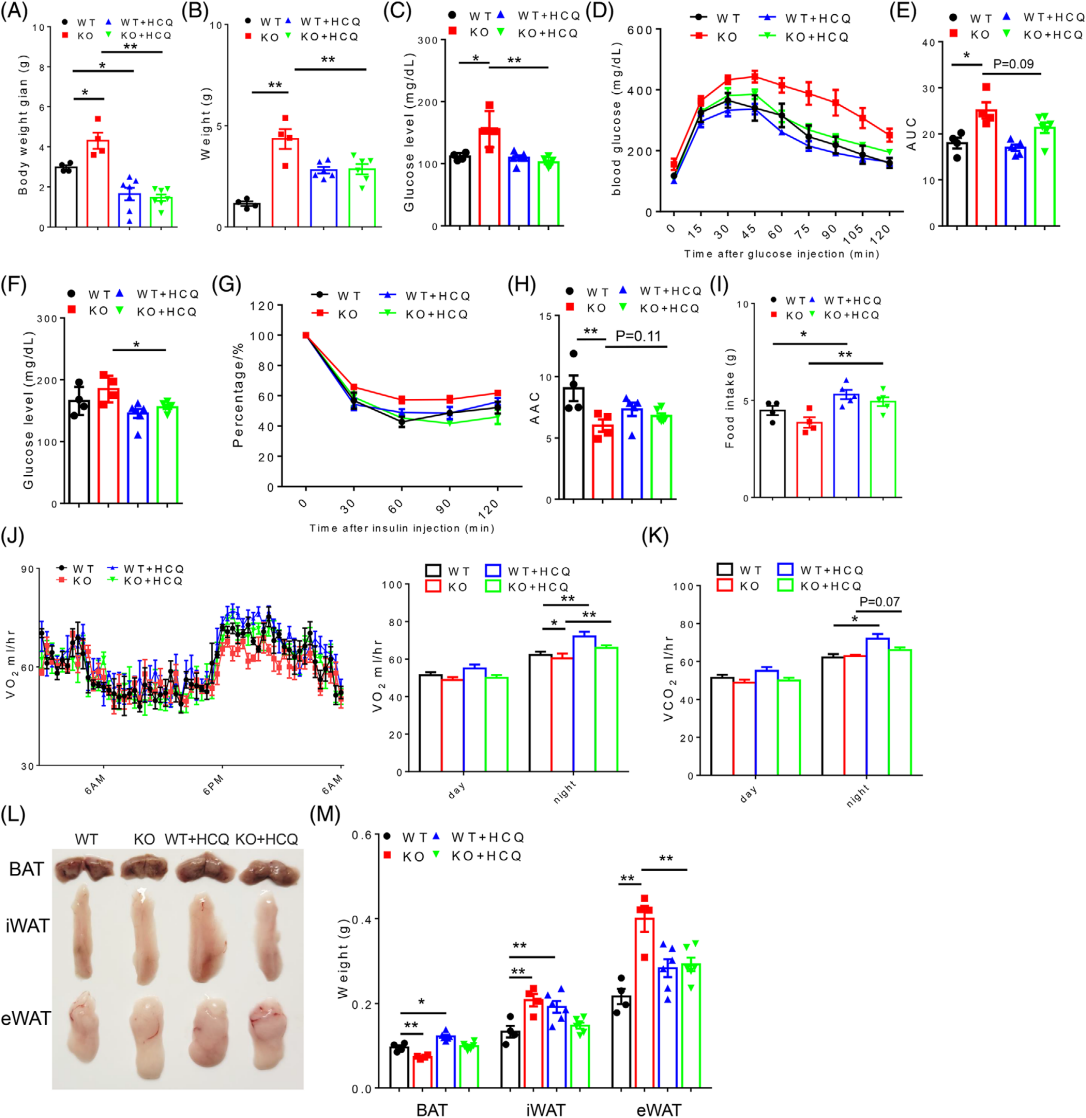
Hydroxychloroquine treatment improves the energy expenditure of Acss3^–/–^ mice. (A, B) Weight gain (A) and body fat mass (B) of male WT and Acss3^–/–^ mice with/without 0.15 mmol/L hydroxychloroquine (HCQ) in the drinking water (named as WT, KO, WT+HCQ and KO+HCQ) after 6 weeks. *N* = 4 pairs of mice with normal drinking and 6 pairs of mice with HCQ water. (C–E) Fasting glucose level (14 h, C), blood glucose concentrations during GTT (D) and AUC (E) of WT and Acss3^–/–^ mice with/without 0.15 mmol/L HCQ treatment. (F–H) Fasting glucose level (6 h, F), blood glucose concentrations during ITT (G) and AAC (H) of WT and Acss3^–/–^ mice with/without 0.15 mmol/L HCQ treatment. (I) Food intake of WT and Acss3^–/–^ mice with/without 0.15 mmol/L HCQ treatment. Data were presented as daily food consumption per mouse from 4 days of each mice. (J, K) O_2_ consumption and average day and night O_2_ consumption (J), and average day and night CO_2_ production (VCO_2_, K) measured by an indirect calorimetry of WT and Acss3^–/–^ mice with/without 0.15 mmol/L HCQ treatment. (L) Representative images of BAT and WAT depots from male mice showing reduction of fat depots of Acss3^–/–^ mice after HCQ treatment. (L) Weights of various BAT and WAT (iWAT, eWAT and asWAT) depots from male WT and Acss3^–/–^ mice with/without 0.15 mmol/L HCQ treatment. Data represent mean ± SEM (*t*‐test: *p < .05, **p < .01)

Using HCQ that is under clinical trial for the prevention of glucose tolerance of T1D patients (NIDDK, NCT03428945), we treated WT and Acss3^–/–^ mice with HCQ (0.15 mmol/L in drinking water) under normal diet and HFD. Increased P62/GAPDH ratio as well as deceased mRNA levels of *Atg5* together indicated the successfully inhibition of autophagy in BAT by HCQ, especially in the Acss3^–/–^ mice (Figure S12A, B). Under normal diet, Acss3^–/–^ mice gained more body weight than WT mice during the 6‐week period (Figure 6A). HCQ treatment significantly reduced the weight gain of both WT and Acss3^–/–^ mice (Figure 6A). The larger body fat mass of Acss3^–/–^ mice was significantly reduced by HCQ treatment (Figure 6B). Interestingly, after HCQ treatment, WT mice had larger body fat mass (Figure 6B). The higher fasting glucose level of Acss3^–/–^ mice was also restored by HCQ treatment (Figure 6C). The impaired glucose tolerance of Acss3^–/–^ mice was also improved by HCQ treatment, though not significant (*p* = 0.09) (Figure 6D, E). Similar results were observed in ITT experiment, where higher fasting glucose levels as well as impaired insulin‐mediated glucose clearance were improved by HCQ treatment (Figure 6F–H). Strikingly, even though both WT and Acss3^–/–^ mice gained less body weight after HCQ treatment, they were both eating more food (Figure 6I). Consistently, Acss3^–/–^ mice had lower levels of O_2_ consumption and CO_2_ production than WT mice (Figure 6J, K). HCQ treatment elevated the O_2_ consumption and CO_2_ production in both WT and Acss3^–/–^ mice (Figure 6J, K). We also inspected various fat depots from the mice (Figure 6L, M). BAT from Acss3^–/–^ mice was smaller and WAT depots were significantly heavier than corresponding fat depots in sex‐matched WT mice (Figure 6L, M), while all WAT depots became smaller after HCQ treatment (Figure 6M). Interestingly, all BAT and iWAT were larger from WT mice after HCQ treatment (Figure 6M).

**FIGURE 7:**
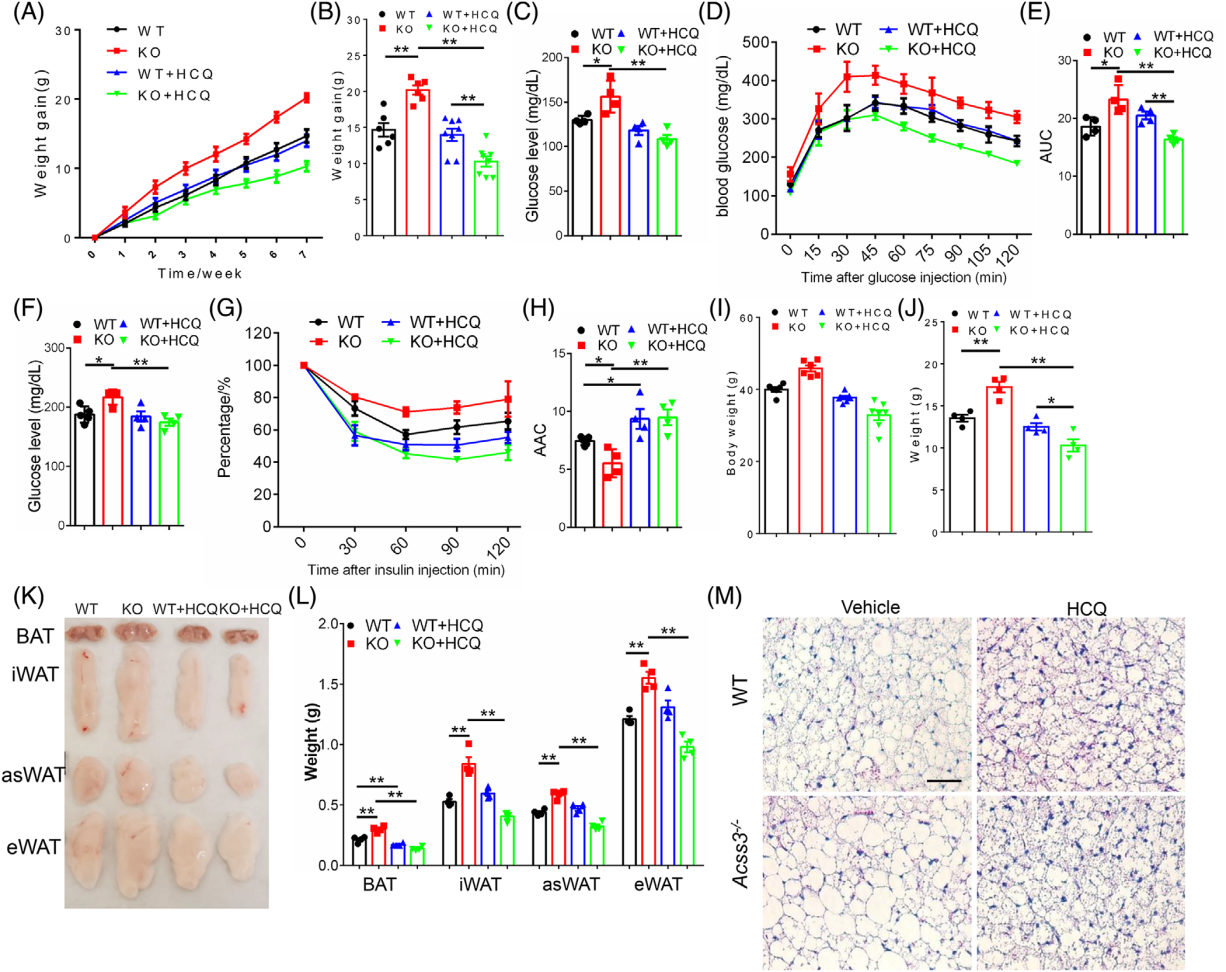
Inhibition of autophagy by hydroxychloroquine normalises fat mass and insulin sensitivity of high‐fat‐diet fed Acss3^–/–^ mice. (A, B) weight gain of male WT and Acss3^–/–^ mice with/without 0.15 mmol/L hydroxychloroquine (HCQ) in the drinking water (named as WT, KO, WT+HCQ and KO+HCQ) during 7 weeks of HFD feeding (A) and after 7 weeks of HFD feeding (B). *N* = 6 pairs of mice with normal drinking water and 8 pairs of mice with HCQ treatment (C–E) Fasting glucose level (14 h, C), blood glucose concentrations during GTT (D) and AUC (E) of WT and Acss3^–/–^ mice with/without 0.15 mmol/L HCQ treatment. (F–H) Fasting glucose level (6 h, F), blood glucose concentrations during ITT (G) and AAC (H) of WT and Acss3^–/–^ mice with/without 0.15 mmol/L HCQ treatment. (I, J) Body weight (I) and fat mass (J) of WT and Acss3^–/–^ mice with/without 0.15 mmol/L HCQ treatment after 10 weeks of HFD feeding. (K) Representative images of BAT and WAT depots from male mice showing reduction of fat depots of Acss3^–/–^ mice after HCQ treatment. (L) Weights of various BAT and WAT (iWAT, eWAT and asWAT) depots from male WT and Acss3^–/–^ mice with/without 0.15 mmol/L HCQ treatment. (M) H&E staining of BAT sections from WT and Acss3^–/–^ mice with/without 0.15 mmol/L HCQ treatment after 10 weeks of HFD feeding. Scale bar: 50 μm. Data represent mean ± SEM (*t*‐test: *p < .05, **p < .01)

When HCQ treatments were applied to HFD‐fed mice, Acss3^–/–^ mice were consistently larger and gain more body weight than WT mice after fed with HFD for 7 weeks (Figure 7A, B). HCQ treatment significantly reduced the weight gain of Acss3^–/–^ mice, but not WT mice (Figure 7A, B). We also tested the glucose tolerance and insulin sensitivity of the mice. Without HCQ treatment, the fasting (14 h) glucose levels of Acss3^–/–^ mice were higher than those of WT mice, while HCQ treatment normalised the fasting glucose levels of Acss3^–/–^ mice (Figure 7C, D). Consistently, control‐treated Acss3^–/–^ mice exhibited impaired glucose tolerance that was rescued by HCQ as indicated by the AUC (Figure 7D, E). HCQ treatment similarly restored the 4 h fasting serum glucose levels of Acss3^–/–^ mice (Figure 7F). In addition, HCQ treatment restored the impaired insulin sensitivity of Acss3^–/–^ mice as indicated by increased AAC (Figure 7G, H). WT mice also exhibited better insulin responses after HCQ treatment (Figure 7G, H). These results demonstrate that HCQ treatment inhibits the weight gain and restores the glucose and insulin sensitivity of *Acss3* KO mice. After fed with HFD for 10 weeks, control‐treated Acss3^–/–^ mice were 5.1 g heavier than WT mice (Figure 7I). Strikingly, HCQ‐treated Acss3^–/–^ mice were 11.7 and 3.5 g lighter than control‐treated Acss3^–/–^ mice and HCQ‐treated WT mice, respectively (Figure 7I). EchoMRI body composition scanning showed that the differences in body weights were mainly due to fat mass change (Figure 7J), Notably, Acss3^–/–^ mice gained 4.0 g more fat mass compared to sex‐matched WT littermates, while HCQ treatment reduced the fat mass of Acss3^–/–^ mice by 10.1 g (Figure 7J). No significant difference was observed in the total fat mass of WT mice with/without HCQ treatment (Figure 7J). We then isolated various fat depots and measured their weights (Figure 7K, L). All Acss3^–/–^ AT depots were significantly heavier than corresponding fat depots in sex‐matched WT mice, while they all became smaller after HCQ treatment (Figure 7L). Interestingly, there were no changes in the weights of WAT depots from WT mice after HCQ treatment, but the BAT mass was significantly reduced (Figure 7L). The LD sizes were obviously larger in Acss3^–/–^ than those of WT BAT after HFD, and they were both reduced by HCQ treatment compared to untreated mice (Figure 7M). In addition, the observed larger and fatty liver in Acss3^–/–^ mice were rescued by HCQ treatment (Figure S12C, D). Collectively, HCQ treatment inhibits fat accumulation in *Acss3* KO mice and ameliorates their metabolic dysfunctions and hepatic steatosis.

## 4 DISCUSSION

**FIGURE 8:**
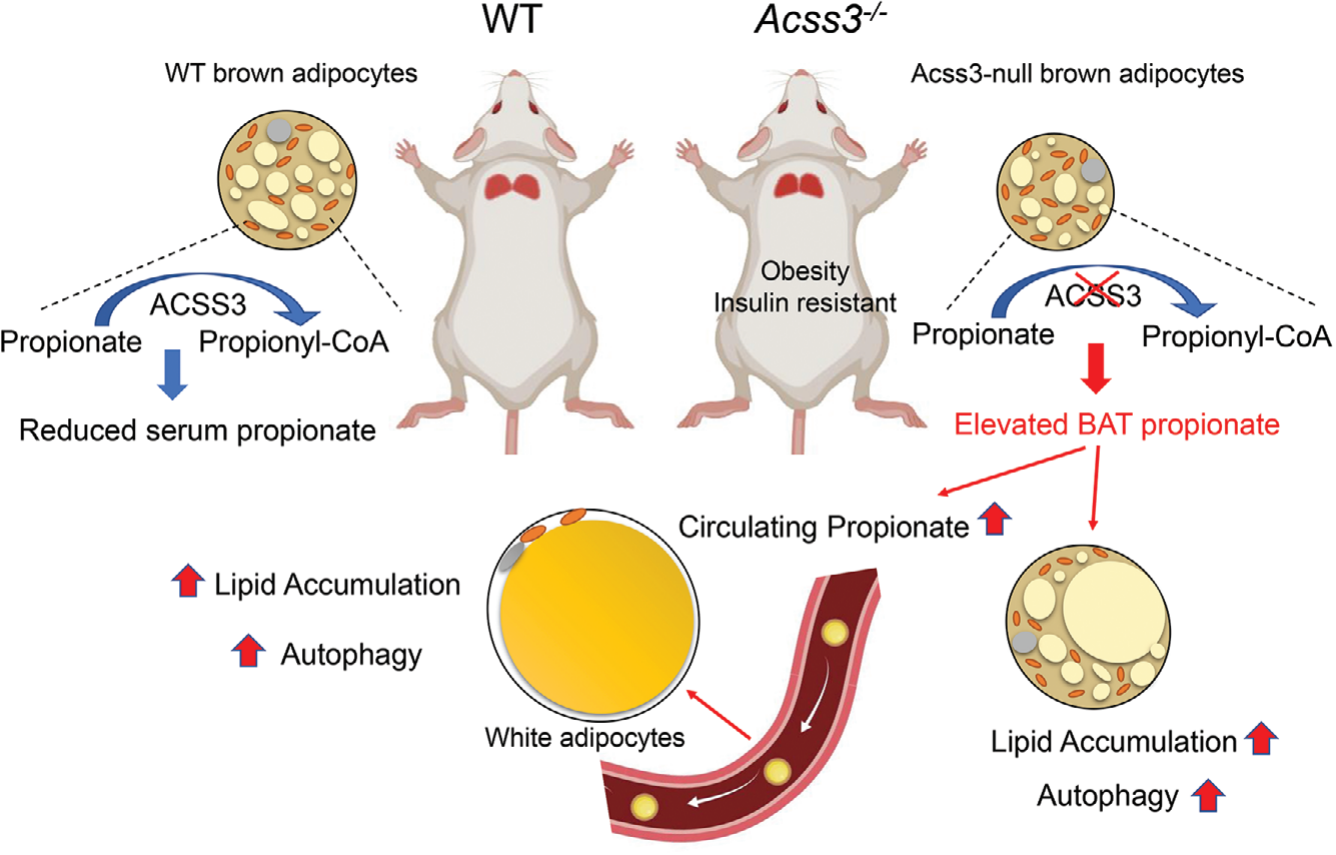
A model depicting ACSS3's role in BAT and propionate metabolism. *Acss3* is highly expressed in BAT and inhibition of *Acss3* disputed BAT adipogenesis, which led to smaller brown adipocytes. Loss of *Acss3* in vivo causes increased fat mass and insulin insensitive in mice, due to elevation its substrate, propionate level in BAT. The elevated propionate BAT propionate promotes the circulating propionate level, which leads to lipid accumulation and autophagy in adipose tissue

Our study demonstrates an in vivo role of ACSS3 in regulating propionate metabolism and autophagy of ATs, thus affecting whole body metabolism and insulin sensitivity (Figure 8). Deletion of *Acss3* leads to loss of BAT mass but increases WAT mass in mice. Interestingly, *Acss3* KO reduces total BAT FAO and causes systemic metabolism defect due to accumulation of its substrate propionate in the serum. We demonstrate that propionate treatment is sufficient to induce autophagy in cultured adipocytes probably through inhibiting mTOR signalling, and Acss3^–/–^ mice exhibit elevated autophagy in their ATs. Pharmacologically inhibition of autophagy by HCQ/wortmannin inhibits propionate‐induced autophagy in vitro, and HCQ treatment ameliorates obesity, hepatic steatosis and insulin resistance of the Acss3^–/–^ mice in vivo. As obesity increases circulating levels of propionate in humans, our results suggest that targeting propionate‐induced autophagy represents a potential therapeutic strategy for obesity and T2D treatment.

We used two specific gRNAs for targeted deletion of exon 2 of *Acss3*, abolishing two coding transcripts of *Acss3*. Thus, our Acss3^–/–^ mice represented an excellent model for studying the function of ACSS3 protein in vivo. Our mice phenotype was consistent with a recent publication that global knockout of *Acss3* slightly increased the body weight of the mice at young age.^41^ However, that study did not analyse the metabolic function of ACSS3 in vivo. To date, most of the studies on *ACSS3* focus on its oncogenic role in human tumours, including gastric cancer, hepatocellular carcinoma and bladder cancer.^17^, ^18^, ^42^ Our observations for the first time extended the function of ACSS3 beyond cancer cells. Specifically, we found that *Acss3* KO caused an obesity‐like phenotype in mice with increased fat mass but smaller BAT. However, the LD sizes were larger in Acss3^–/–^ BAT at 6‐month‐old, which indicates that there were fewer BAs. In contrast, even though BAT mass of Acss3^–/–^ mice was already reduced at 2‐month‐old, the sizes of LD were smaller at this age. These observations indicate that the function of ACSS3 in BAs might be stage‐specific. The smaller BAs and LDs at younger Acss3^–/–^ BAT may be due to BA developmental or differentiation defects. In addition, we found that both *Acss3* KD or KO BA cells had less LD after differentiation. However, the mechanism underlying the effect of ACSS3 on BA differentiation remains unknown and warrants future investigation. While the larger LDs in *Acss3*‐null mature BA from 6‐month‐old mice may be due to the autophagy‐induced lipid accumulation or secondary effects of global metabolic defects. Stage‐specific function of other genes has also been reported in BAs. For example, loss of *Prdm16* in *Myf5*‐positive cells does not affect embryonic BAT development but disrupts BA identity and function in young mice and during aging.^29^, ^43^, ^44^ Furthermore, although we found that *Acss3* was predominantly expressed in BAT, loss of ACSS3 function in other cell types expressing low levels of Acss3 might have also contributed to the phenotypes of Acss3^–/–^ mice, especially the propionate accumulation. Coincidently, ACSS3 has been reported to play a role in rat liver cell and mouse prostate.^16^, ^41^ Thus, future study using UCP1^Cre^‐driven *Acss3* knockout in BAs will pinpoint the function of ACSS3 in BAs.

Of the three ACSSs, ACSS1 and ACSS2 are well characterised as acetyl‐CoA synthetases in mitochondria and cytosol, respectively.^45^, ^46^, ^47^ We for the first time characterised that ACSS3 was a mitochondrial inner membrane protein. The activation of fatty acids is a two‐step reaction.^5^ The transmembrane structure ACSS3 implies that the two‐step propionate activation that requires different functional domain of ACSS3 may occur in different compartments of mitochondria. In addition, it would be interesting to investigate in future if ACSS3 serves any additional functions within and outside mitochondria. For example, ACSS2 is recently found to function in the nuclear as a chromatin‐bound transcriptional coactivator that directly regulates histone acetylation and gene expression.^48^, ^49^ Indeed, we also observed some ACSS3 signal outside of Mito‐tracker in both undifferentiated and differentiated BAs. It is possible that ACSS3 might be located in the nuclear or other organelles such as endoplasmic reticulum (ER). A recent study has revealed a role of ACSS3 in LD degradation via interacting with and stabilising perilipin 4.^41^ Studies have also identified histone propionylation as an important modification in regulating chromatin structure and function,^50^, ^51^, ^52^ suggesting a potential role of ACSS3 in the nucleus. Considering that there are two isoforms of ACSS3 protein (and we only detected the longer isoform in mitochondria), future studies should explore the function of different ACSS3 isoforms.

Our study identifies a unique substrate preference of ACSS3 for propionate as previously reported,^16^ and *Acss3* KO elevated propionate in both BAT and serum. Emerging evidence has pointed out the importance of propionate produced by colonic microbiota in metabolic health.^53^, ^54^, ^55^ Interestingly, we found that the impaired energy expenditure of Acss3^–/–^ mice was only found at night‐time (6 pm to 6 am) when mice actively feed. This observation is consistent with the notion that propionate is mainly released from food by gut bacteria after feeding. In addition, Acss3^–/–^ mice were less active at night, which might be a consequence of propionate accumulation associated with higher food intake during night‐time. Thus, it would also be interesting to compare the diurnal propionate levels in Acss3^–/–^ mice. Propionate has been reported to promote adipogenesis and fat accumulation of WAs via activation of GPR43 in vitro.^56^, ^57^ Dietary supplementation of propionate also shows anti‐obese activity through inducing expressions of GPR43 and GPR41.^58^ However, in a recent study, it was reported that propionate promotes the lipid accumulation in adipocytes and causes a gradual weight gain and insulin resistance in mice, while serum propionate level is highly correlated with human obesity.^11^ The results are consistent with our *Acss3* KO mice that had elevated propionate in the serum, associated with an obese phenotype. In addition, we found that propionate promoted the expression of adipogenic markers and lipid accumulation of cultured WAs in vitro, and *Acss3‐*null adipocytes also formed larger LDs. These observations suggest that propionate accumulation is a main metabolic problem, while dietary supplementation of propionate can be metabolically beneficial as fuel source if it is timely activated. Propionate can also activate GPR43 in adipocytes to exert metabolic effects. Taken together, these results corroborate a critical requirement for ACSS3‐mediated propionate metabolism in the maintenance of adipocyte homeostasis.

Autophagy plays vital roles in the differentiation and metabolism of both WAs and BAs.^24^, ^25^, ^26^ Our *Acss3* KO mice exhibited elevated autophagy in ATs, increased WAT mass and impaired insulin sensitivity that were exacerbated by HFD. It has been reported that deregulation of autophagy promotes metabolic disorders such as obesity and diabetes mellitus.^59^ Autophagy is regulated by multiple signalling molecules, including the nutrient sensing mTOR kinase. During the development of obesity, imbalanced food intake and energy expenditure lead to alteration of autophagy due to mTOR signalling.^59^ In addition, propionate has been shown to induce autophagy through downregulation of mTOR signalling.^31^ We also found that propionate treatment in adipocytes suppressed the phosphorylation of 4E‐BP1, a key downstream effector of mTOR activity.^60^ We speculate that deletion of *Acss3* promotes propionate accumulation, which changes the nutrient status of adipocytes, and subsequentially induces autophagy through inhibition of mTOR. These phenotypes were consistent with those of adipocyte‐specific *Atg7* KO mice, where loss of autophagic function decreases WAT mass, enhances insulin sensitivity and protects the mice from HFD‐induced obesity.^24^, ^25^ Interestingly, the enhanced autophagy did not change UCP1 nor mitochondrial protein levels in Acss3^–/–^ BAT. Similar results have also been reported in BA‐specific *Atg12* KO mice, in which inhibition of autophagy by *Atg12* KO in BAT does not change the protein level of UCP1 and mitochondrial proteins.^26^ Instead, genetic and pharmacological inhibition of autophagy retains beige adipocyte function through blocking autophagy‐dependent mitochondria clearance.^26^ We observed possibly reduced beige adipogenesis in Acss3^–/–^ iWAT during cold‐induced thermogenesis. Although rectal temperature of Acss3^–/–^ mice dropped faster within 2 h of acute challenge, the mice had comparable capacity for maintaining the temperature later on, suggesting that the smaller BAT or muscle shivering response is functionally efficient for thermogenesis. However, like BCAA acts as an energy source in BAT mitochondria during thermogenesis,^61^ there is possibility that propionate may also enter the TCA cycle to produce energy. But how BAT utilise propionate needs to be further investigated.

Recent studies have shown that human BAT is decreased with BMI and adiposity,^62^, ^63^, ^64^ whereas autophagy is upregulated in AT of obese subjects with more visceral fat distribution.^22^, ^23^, ^65^, ^66^ Consistent with these findings, the propionate‐induced autophagy in adipocytes of *Acss3* KO mice contributed to obesity, insulin resistance and reduced BAT mass. By analysing previously published data, we found that propionate concentrations were increased in the faeces and serum of obese and T2D subjects.^36^, ^37^ On the other hand, *ACSS3* expression in WAT was decreased with obesity^38^ and impaired insulin sensitivity.^39^ These data provide compelling evidence that the increased propionate production and impaired ACSS3‐mediated propionate metabolism are highly relevant to obesity and T2D. By inhibition of autophagy using HCQ treatment, the fat mass gain and impaired insulin sensitivity of Acss3^–/–^ mice were normalised. Interestingly, the effect of HCQ was more profound in *Acss3* KO than WT mice, suggesting that autophagy is the mainly driver of fat accumulation in *Acss3* KO mice. However, we could not exclude the other potential effects of HCQ on non‐ATs. The observation that HCQ treatment inhibited the fat accumulation of BAT and liver of WT mice suggests that targeting autophagy represents a promising therapeutic strategy that could be translated into the clinical treatment of obesity and diabetes patients.^67^ Supporting this, an ongoing clinical trial is investigating the effect of HCQ in the prevention of glucose tolerance of T1D patients (NIDDK, NCT03428945). Compared to the HCQ dosage used in our study, one of the studies applied 4‐fold higher HCQ concentration to treat the mice continuously for 17 weeks, resulting in a stronger anti‐obesity effect.^33^ Our data demonstrate that lower doses of HCQ treatment are efficient in improving the insulin sensitivity of HFD‐fed mice even without reducing WAT mass. Future studies combining nano‐materials for targeted and controlled drug delivery could further improve the efficiency HCQ treatment.^68^, ^69^

## CONFLICT OF INTEREST

The authors declare no conflict of interest.

## Supporting information

Supporting Information

Supporting Information

## References

[1] Galisteo M , Duarte J , Zarzuelo A . Effects of dietary fibers on disturbances clustered in the metabolic syndrome. J Nutr Biochem. 2008;19(2):71‐84. 17618108 10.1016/j.jnutbio.2007.02.009

[2] Ren J , Wu NN , Wang S , Sowers JR , Zhang Y . Obesity cardiomyopathy: evidence, mechanisms and therapeutic implications. Physiol Rev. 2021;101(4):1745‐1807. 10.1152/physrev.00030.2020 PMC8422427 33949876

[3] Karpe F , Dickmann JR , Frayn KN . Fatty acids, obesity, and insulin resistance: time for a reevaluation. Diabetes. 2011;60(10):2441‐2449. 21948998 10.2337/db11-0425 PMC3178283

[4] Lottenberg AM , da Silva Afonso M , Lavrador MSF , Machado RM , Nakandakare ER . The role of dietary fatty acids in the pathology of metabolic syndrome. J Nutr Biochem. 2012;23(9):1027‐1040. 22749135 10.1016/j.jnutbio.2012.03.004

[5] Watkins PA , Maiguel D , Jia Z , Pevsner J . Evidence for 26 distinct acyl‐coenzyme A synthetase genes in the human genome. J Lipid Res. 2007;48(12):2736‐2750. 17762044 10.1194/jlr.M700378-JLR200

[6] Watkins PA . Fatty acid activation. Prog Lipid Res. 1997;36(1):55‐83. 9373621 10.1016/s0163-7827(97)00004-0

[7] Ellis JM , Frahm JL , Li LO , Coleman RA . Acyl‐coenzyme A synthetases in metabolic control. Curr Opin Lipidol. 2010;21(3):212. 20480548 10.1097/mol.0b013e32833884bb PMC4040134

[8] Yan S , Yang X , Liu H , Fu N , Ouyang Y , Qing K . Long‐chain acyl‐CoA synthetase in fatty acid metabolism involved in liver and other diseases: an update. World J Gastroenterol. 2015;21(12):3492. 25834313 10.3748/wjg.v21.i12.3492 PMC4375570

[9] Chambers ES , Preston T , Frost G , Morrison DJ . Role of gut microbiota‐generated short‐chain fatty acids in metabolic and cardiovascular health. Curr Nutr Rep. 2018;7(4):198‐206. 30264354 10.1007/s13668-018-0248-8 PMC6244749

[10] Byrne C , Chambers E , Morrison D , Frost G . The role of short chain fatty acids in appetite regulation and energy homeostasis. Int J Obes. 2015;39(9):1331‐1338. 10.1038/ijo.2015.84 PMC4564526 25971927

[11] Tirosh A , Calay ES , Tuncman G , et al. The short‐chain fatty acid propionate increases glucagon and FABP4 production, impairing insulin action in mice and humans. Sci Transl Med. 2019;11(489):eaav0120. 31019023 10.1126/scitranslmed.aav0120

[12] Georgiadi A , Kersten S . Mechanisms of gene regulation by fatty acids. Adv Nutr. 2012;3(2):127‐134. 22516720 10.3945/an.111.001602 PMC3648713

[13] Hara T , Kashihara D , Ichimura A , Kimura I , Tsujimoto G , Hirasawa A . Role of free fatty acid receptors in the regulation of energy metabolism. Biochim Biophys Acta. 2014;1841(9):1292‐1300. 24923869 10.1016/j.bbalip.2014.06.002

[14] Gao X , Lin S , Ren F , et al. Acetate functions as an epigenetic metabolite to promote lipid synthesis under hypoxia. Nat Commun. 2016;7(1):1‐14. 10.1038/ncomms11960 PMC4931325 27357947

[15] Comerford SA , Huang Z , Du X , et al. Acetate dependence of tumors. Cell. 2014;159(7):1591‐1602. 25525877 10.1016/j.cell.2014.11.020 PMC4272450

[16] Yoshimura Y , Araki A , Maruta H , Takahashi Y , Yamashita H . Molecular cloning of rat acss3 and characterization of mammalian propionyl‐CoA synthetase in the liver mitochondrial matrix. J Biochem. 2017;161(3):279‐289. 28003429 10.1093/jb/mvw067

[17] Chang W , Cheng W , Cheng B , et al. Mitochondrial acetyl‐CoA synthetase 3 is biosignature of gastric cancer progression. Cancer Med. 2018;7(4):1240‐1252. 29493120 10.1002/cam4.1295 PMC5911630

[18] Zhang J , Duan H , Feng Z , Han X , Gu C . Acetyl‐CoA synthetase 3 promotes bladder cancer cell growth under metabolic stress. Oncogenesis. 2020;9(5):1‐10. 32398651 10.1038/s41389-020-0230-3 PMC7217873

[19] Choi AM , Ryter SW , Levine B . Autophagy in human health and disease. N Engl J Med. 2013;368(7):651‐662. 23406030 10.1056/NEJMra1205406

[20] Rabinowitz JD , White E . Autophagy and metabolism. Science. 2010;330(6009):1344‐1348. 21127245 10.1126/science.1193497 PMC3010857

[21] Kim KH , Lee MS . Autophagy – a key player in cellular and body metabolism. Nat Rev Endocrinol. 2014;10(6):322. 24663220 10.1038/nrendo.2014.35

[22] Jansen H , Van Essen P , Koenen T , et al. Autophagy activity is up‐regulated in adipose tissue of obese individuals and modulates proinflammatory cytokine expression. Endocrinology. 2012;153(12):5866‐5874. 23117929 10.1210/en.2012-1625

[23] Kovsan J , Blüher M , Tarnovscki T , et al. Altered autophagy in human adipose tissues in obesity. J Clin Endocrinol Metab. 2011;96(2):E268‐E77. 21047928 10.1210/jc.2010-1681

[24] Singh R , Xiang Y , Wang Y , et al. Autophagy regulates adipose mass and differentiation in mice. J Clin Invest. 2009;119(11):3329‐3339. 19855132 10.1172/JCI39228 PMC2769174

[25] Zhang Y , Goldman S , Baerga R , Zhao Y , Komatsu M , Jin S . Adipose‐specific deletion of autophagy‐related gene 7 (atg7) in mice reveals a role in adipogenesis. Proc Natl Acad Sci USA. 2009;106(47):19860‐19865. 19910529 10.1073/pnas.0906048106 PMC2785257

[26] Altshuler‐Keylin S , Shinoda K , Hasegawa Y , et al. Beige adipocyte maintenance is regulated by autophagy‐induced mitochondrial clearance. Cell Metab. 2016;24(3):402‐419. 27568548 10.1016/j.cmet.2016.08.002 PMC5023491

[27] Wolpin BM , Rubinson DA , Wang X , et al. Phase II and pharmacodynamic study of autophagy inhibition using hydroxychloroquine in patients with metastatic pancreatic adenocarcinoma. Oncologist. 2014;19(6):637. 24821822 10.1634/theoncologist.2014-0086 PMC4041680

[28] Tang Y , Chen Y , Jiang H , Nie D . Short‐chain fatty acids induced autophagy serves as an adaptive strategy for retarding mitochondria‐mediated apoptotic cell death. Cell Death Differ. 2011;18(4):602‐618. 20930850 10.1038/cdd.2010.117 PMC3020988

[29] Harms MJ , Ishibashi J , Wang W , et al. Prdm16 is required for the maintenance of brown adipocyte identity and function in adult mice. Cell Metab. 2014;19(4):593‐604. 24703692 10.1016/j.cmet.2014.03.007 PMC4012340

[30] Chen Y , Ikeda K , Yoneshiro T , et al. Thermal stress induces glycolytic beige fat formation via a myogenic state. Nature. 2019;565(7738):180‐185. 30568302 10.1038/s41586-018-0801-z PMC6328316

[31] Park J , Kim M , Kang SG , et al. Short‐chain fatty acids induce both effector and regulatory T cells by suppression of histone deacetylases and regulation of the mTOR–S6K pathway. Mucosal Immunol. 2015;8(1):80‐93. 24917457 10.1038/mi.2014.44 PMC4263689

[32] Yue F , Bi P , Wang C , et al. Pten is necessary for the quiescence and maintenance of adult muscle stem cells. Nature Commun. 2017;8(1):1‐13. 28094257 10.1038/ncomms14328 PMC5247606

[33] Qiao X , Zhou Z , Niu R , et al. Hydroxychloroquine improves obesity‐associated insulin resistance and hepatic steatosis by regulating lipid metabolism. Frontiers Pharmacol. 2019;10:855. 10.3389/fphar.2019.00855 PMC6689966 31427967

[34] Song A , Dai W , Jang MJ , et al. Low‐ and high‐thermogenic brown adipocyte subpopulations coexist in murine adipose tissue. J Clin Invest. 2020;130(1):247‐257. 31573981 10.1172/JCI129167 PMC6934193

[35] Burl RB , Ramseyer VD , Rondini EA , Pique‐Regi R , Lee Y‐H , Granneman JG . Deconstructing adipogenesis induced by β3‐adrenergic receptor activation with single‐cell expression profiling. Cell Metab. 2018;28(2):300‐309. 29937373 10.1016/j.cmet.2018.05.025 PMC6082711

[36] Zhao L , Lou H , Peng Y , Chen S , Fan L , Li X . Elevated levels of circulating short‐chain fatty acids and bile acids in type 2 diabetes are linked to gut barrier disruption and disordered gut microbiota. Diabetes Res Clin Pract. 2020;169:108418. 32891692 10.1016/j.diabres.2020.108418

[37] Schwiertz A , Taras D , Schäfer K , et al. Microbiota and SCFA in lean and overweight healthy subjects. Obesity. 2010;18(1):190‐195. 19498350 10.1038/oby.2009.167

[38] Lee Y , Nair S , Rousseau E , et al. Microarray profiling of isolated abdominal subcutaneous adipocytes from obese vs non‐obese Pima Indians: increased expression of inflammation‐related genes. Diabetologia. 2005;48(9):1776‐1783. 16059715 10.1007/s00125-005-1867-3 PMC1409820

[39] Hardy OT , Perugini RA , Nicoloro SM , et al. Body mass index‐independent inflammation in omental adipose tissue associated with insulin resistance in morbid obesity. Surg Obes Relat Dis. 2011;7(1):60‐67. 20678967 10.1016/j.soard.2010.05.013 PMC2980798

[40] Cheng X , Xie Y , Zhou B , Huang N , Farfel‐Becker T , Sheng Z . Revisiting LAMP1 as a marker for degradative autophagy‐lysosomal organelles in the nervous system. Autophagy. 2018;14(8):1472‐1474. 29940787 10.1080/15548627.2018.1482147 PMC6103665

[41] Zhou L , Song Z , Hu J , et al. ACSS3 represses prostate cancer progression through downregulating lipid droplet‐associated protein PLIN3. Theranostics. 2021;11(2):841‐860. 33391508 10.7150/thno.49384 PMC7738848

[42] Bidkhori G , Benfeitas R , Klevstig M , et al. Metabolic network‐based stratification of hepatocellular carcinoma reveals three distinct tumor subtypes. Proc Natl Acad Sci USA. 2018;115(50):E11874. 30482855 10.1073/pnas.1807305115 PMC6294939

[43] Seale P , Bjork B , Yang W , et al. PRDM16 controls a brown fat/skeletal muscle switch. Nature. 2008;454(7207):961‐967. 18719582 10.1038/nature07182 PMC2583329

[44] Wang W , Ishibashi J , Trefely S , et al. A PRDM16‐driven metabolic signal from adipocytes regulates precursor cell fate. Cell Metab. 2019;30(1):174‐189. e5. 31155495 10.1016/j.cmet.2019.05.005 PMC6836679

[45] Schwer B , Bunkenborg J , Verdin RO , Andersen JS , Verdin E . Reversible lysine acetylation controls the activity of the mitochondrial enzyme acetyl‐CoA synthetase 2. Proc Natl Acad Sci USA. 2006;103(27):10224‐10229. 16788062 10.1073/pnas.0603968103 PMC1502439

[46] Fujino T , Kondo J , Ishikawa M , Morikawa K , Yamamoto TT . Acetyl‐CoA synthetase 2, a mitochondrial matrix enzyme involved in the oxidation of acetate. J Biol Chem. 2001;276(14):11420‐11426. 11150295 10.1074/jbc.M008782200

[47] Ikeda Y , Yamamoto J , Okamura M , et al. Transcriptional regulation of the murine acetyl‐CoA synthetase 1 gene through multiple clustered binding sites for sterol regulatory element‐binding proteins and a single neighboring site for Sp1. J Biol Chem. 2001;276(36):34259‐34269. 11435428 10.1074/jbc.M103848200

[48] Mews P , Donahue G , Drake AM , Luczak V , Abel T , Berger SL . Acetyl‐CoA synthetase regulates histone acetylation and hippocampal memory. Nature. 2017;546(7658):381‐386. 28562591 10.1038/nature22405 PMC5505514

[49] Li X , Yu W , Qian X , et al. Nucleus‐translocated ACSS2 promotes gene transcription for lysosomal biogenesis and autophagy. Mol Cell. 2017;66(5):684‐697. e9. 28552616 10.1016/j.molcel.2017.04.026 PMC5521213

[50] Chen Y , Sprung R , Tang Y , et al. Lysine propionylation and butyrylation are novel post‐translational modifications in histones. Mol Cell Proteomics. 2007;6(5):812‐819. 17267393 10.1074/mcp.M700021-MCP200 PMC2911958

[51] Kebede AF , Nieborak A , Shahidian LZ , et al. Histone propionylation is a mark of active chromatin. Nat Struct Mol Biol. 2017;24(12):1048. 29058708 10.1038/nsmb.3490

[52] Han Z , Wu H , Kim S , et al. Revealing the protein propionylation activity of the histone acetyltransferase MOF (males absent on the first). J Biol Chem. 2018;293(9):3410‐3420. 29321206 10.1074/jbc.RA117.000529 PMC5836141

[53] Louis P , Flint HJ . Formation of propionate and butyrate by the human colonic microbiota. Environ Microbiol. 2017;19(1):29‐41. 27928878 10.1111/1462-2920.13589

[54] Bartolomaeus H , Balogh A , Yakoub M , et al. Short‐chain fatty acid propionate protects from hypertensive cardiovascular damage. Circulation. 2019;139(11):1407‐1421. 30586752 10.1161/CIRCULATIONAHA.118.036652 PMC6416008

[55] Koh A , De Vadder F , Kovatcheva‐Datchary P , Bäckhed F . From dietary fiber to host physiology: short‐chain fatty acids as key bacterial metabolites. Cell. 2016;165(6):1332‐1345. 27259147 10.1016/j.cell.2016.05.041

[56] Ge H , Li X , Weiszmann J , et al. Activation of G protein‐coupled receptor 43 in adipocytes leads to inhibition of lipolysis and suppression of plasma free fatty acids. Endocrinology. 2008;149(9):4519‐4526. 18499755 10.1210/en.2008-0059

[57] Dewulf EM , Cani PD , Neyrinck AM , et al. Inulin‐type fructans with prebiotic properties counteract GPR43 overexpression and PPARγ‐related adipogenesis in the white adipose tissue of high‐fat diet‐fed mice. J Nutr Biochem. 2011;22(8):712‐722. 21115338 10.1016/j.jnutbio.2010.05.009

[58] Lu Y , Fan C , Li P , Lu Y , Chang X , Qi K . Short chain fatty acids prevent high‐fat‐diet‐induced obesity in mice by regulating G protein‐coupled receptors and gut microbiota. Sci Rep. 2016;6(1):1‐13. 27892486 10.1038/srep37589 PMC5124860

[59] Zhang Y , Sowers JR , Ren J . Targeting autophagy in obesity: from pathophysiology to management. Nat Rev Endocrinol. 2018;14(6):356‐376. 29686432 10.1038/s41574-018-0009-1

[60] Inoki K , Zhu T , Guan KL . TSC2 mediates cellular energy response to control cell growth and survival. Cell. 2003;115(5):577‐590. 14651849 10.1016/s0092-8674(03)00929-2

[61] Yoneshiro T , Wang Q , Tajima K , Matsushita M , et al. BCAA catabolism in brown fat controls energy homeostasis through slc25a44. Nature. 2019;572(7771). 10.1038/s41586-019-1503-x PMC6715529 31435015

[62] Cypess AM , Lehman S , Williams G , et al. Identification and importance of brown adipose tissue in adult humans. N Engl J Med. 2009;360(15):1509‐1517. 19357406 10.1056/NEJMoa0810780 PMC2859951

[63] Saito M , Okamatsu‐Ogura Y , Matsushita M , et al. High incidence of metabolically active brown adipose tissue in healthy adult humans: effects of cold exposure and adiposity. Diabetes. 2009;58(7):1526‐1531. 19401428 10.2337/db09-0530 PMC2699872

[64] van Marken Lichtenbelt WD , Vanhommerig JW , Smulders NM , et al. Cold‐activated brown adipose tissue in healthy men. N Engl J Med. 2009;360(15):1500‐1508. 19357405 10.1056/NEJMoa0808718

[65] Kosacka J , Kern M , Klöting N , et al. Autophagy in adipose tissue of patients with obesity and type 2 diabetes. Mol Cell Endocrinol. 2015;409:21‐32. 25818883 10.1016/j.mce.2015.03.015

[66] Nunez C , Rodrigues V , Gomes F , et al. Defective regulation of adipose tissue autophagy in obesity. Int J Obes. 2013;37(11):1473‐1480. 10.1038/ijo.2013.27 23478428

[67] Wondafrash DZ , Desalegn TZ , Yimer EM , Tsige AG , Adamu BA , Zewdie KA . Potential effect of hydroxychloroquine in diabetes mellitus: a systematic review on preclinical and clinical trial studies. J Diabetes Res. 2020;2020:5214751. 10.1155/2020/5214751 PMC7064866 32190699

[68] Won YW , Adhikary PP , Lim KS , Kim HJ , Kim JK , Kim Y‐H . Oligopeptide complex for targeted non‐viral gene delivery to adipocytes. Nat Mater. 2014;13(12):1157‐1164. 25282508 10.1038/nmat4092

[69] Huang D , Narayanan N , Cano‐Vega MA , et al. Nanoparticle‐mediated inhibition of Notch signaling promotes mitochondrial biogenesis and reduces subcutaneous adipose tissue expansion in pigs. iScience. 2020;23:101167. 32480124 10.1016/j.isci.2020.101167 PMC7262558

