# Supplementary material for: ACSS3 in brown fat drives propionate catabolism and its deficiency leads to autophagy and systemic metabolic dysfunction
Source: Clin Transl Med. 2022 Feb 20;12(2):e665. doi: 10.1002/ctm2.665 (PMC8858619; doi:10.1002/ctm2.665)
Supplement: Supplementary file 1 — Supporting Information [file CTM2-12-e665-s002.docx]

**ACSS3 in brown fat drives propionate catabolism and its deficiency leads to autophagy and systemic metabolic dysfunction**

Zhihao Jia^1^, Xiyue Chen^1^, Jingjuan Chen^1^, Lijia Zhang^1^, Stephanie N. Oprescu^1,2^, Nanjian Luo^1^, Yan Xiong^1^, Feng Yue^1^, Shihuan Kuang^1, 2, 3, #^

^1^Department of Animal Sciences, Purdue University, West Lafayette, Indiana, 47906, USA.

^2^Department of Biological Sciences, ^3^Center for Cancer Research, Purdue University, West Lafayette, Indiana, 47906, USA.

**This file includes：**

Supplementary Figure Legends S1 to S6

Supplementary references

**Supplementary Figure Legends**

**Figure S1. *Acss3* expression in BAT.**

(A) UMAP embedding of scRNA-seq data of BAT colored by different clusters (left) to simplify visualization and relative *Ucp1* mRNA levels (right). (C) Violin plots grouped by meta-clusters demonstrate relative mRNA levels of *Adipoq*, *Cebpα*, *Plin1*, *Pdgfrα*, *Ucp1* and *Acss3* in each clusters. The scRNA-seq data was retrieved from NCBI Gene Expression Omnibus database (accession number GSE125269).

**Figure S2. *Acss3* expression in eWAT after CL treatment.**

(A) UMAP embedding of scRNA-seq data of eWAT from control and CL-316,243 treatment colored by different clusters to simplify visualization. (B) Violin plots grouped by meta-clusters demonstrate relative mRNA levels of *Adipoq, Pdgfrα*, *Ucp1* and *Acss3* in each clusters. (C) Relative *Adipoq*, *Pdgfrα*, *Ucp1* and *Acss3* mRNA levels showing the specific expression of *Ucp1* from cluster 4. (D) UMAP embedding of scRNA-seq data of cluster 4 from CL-316,243 treatment colored by different clusters to simplify visualization. (E) Violin plots grouped by meta-clusters demonstrate relative mRNA levels of *Adipoq, Pdgfrα*, *Ucp1* and *Acss3* in each clusters. The scRNA-seq data was retrieved from the Sequence Read Archive (SRA; https://www.ncbi.nlm.nih.gov/sra/) (SRA: SRP145475).

**Figure S3. *Acss3* expression is transcriptionally controlled by C/EBPα.**

(A) Schematic of Pgl3-*Acss3* luciferase reporter driven by a 2 kb-genomic sequence upstream of *Acss3* transcription start site containing two consensus C/EBPα binding sites and one PPARγ binding site. (B) Luciferase assay of co-transfected with PPARγ*,* C/EBPα, Pgl3-basic and Pgl3-*Acss3* promoter (Pgl3-*Acss3*-P) in 293 T cells (n = 6, 3 biological replicates for each independent experiment). Data represent mean ± s.e.m. (t-test: ** P<0.01).​

**Figure S4. Deletion of *Acss3* reduces mass of BAT, but not other tissues.**

(A) Representative images of BAT and WAT depots from male mice showing reduced BAT mass and increased WAT mass of Acss3^-/-^ mice at 6-month-old. (B, C) weights of various muscle tissues (B), liver, heart and kidney (C) of WT and Acss3^-/-^ mice at 6-month-old, N=6 pairs mice. Data represent mean±s.e.m. (t-test: **P<0.01).

**Figure S5. Deletion of *Acss3* reduces mass of BAT, and inhibits BAT differentiation.**

(A, B) Body weight (A) and weights of various fat depots (B) of WT and Acss3^-/-^ mice at 2-month-old, N=3 pairs mice. (C) Representative images (upper) and H&E staining (lower) of BAT from WT and Acss3^-/-^ mice at 2MO, scale bar: 50 μm. (D) Relative cell areas of from WT and  Acss3^-/-^ BAT. (E, F) Body weight (E) and weights of various fat depots (F) of WT and Acss3^-/-^ mice at 6-week-old, N=4 pairs mice. (G) Representative images (upper) and H&E staining (lower) of BAT from WT and Acss3^-/-^ mice at 6-week-old, scale bar: 50 μm. (H) Relative cell areas of from WT and Acss3^-/-^ BAT. (H) H&E staining (lower) of eWAT from WT and Acss3^-/-^ mice at 6-week-old, scale bar: 100 μm. (J, K) Body weight (J) and weights of various fat depots (K) of WT and Acss3^-/-^ mice at postnatal day 7 (P7), N=3 pairs mice. (L) Representative images (upper) and H&E staining (lower) of BAT from WT and Acss3^-/-^ mice at P7, scale bar: 50 μm. (M) Relative cell areas of from WT and Acss3^-/-^ BAT. (N) H&E staining (lower) of eWAT from WT and Acss3^-/-^ mice at P7, scale bar: 100 μm. (O) Western-blot analysis of UCP1 and FABP4 from BAT lysate of WT and Acss3^-/-^ mice at P7. (P) Representative images of bodipy and DAPI staining of SVF preadipocytes from WT and Acss3^-/-^ BAT after 8-day of differentiation, scale bar: 50 μm. Data represent mean±s.e.m. (t-test: *P<0.05, **P<0.01).

**Figure S6. Knockdown of *Acss3* by shRNA inhibits brown adipocyte differentiation.**

(A) Knockdown (KD) efficiency of two independent lentiviral shRNAs on *Acss3* expression. (B) Representative images of bodipy and DAPI staining of shRNA1 and shRNA2 lentivirus stable Acss3 KD BAT cell lines (sh1 and sh2) after 6-day of differentiation, scale bar: 50 μm. (C) Total cell counts of sh1 and sh2. (D, E) Relative mRNA levels of adipogenesis (D) and browning (E) genes in sh1 and sh2 after 6-day of differentiation. (F, G) Protein levels (F) and ratios to GAPDH (G) of PLIN1, PGC1α, PPARγ and C/EBPα in sh1 and sh2 after 6-day of differentiation. (H, I) Protein levels (H) and ratios to GAPDH (I) of mitochondrial OXPHOS complexes, including ATP5A, UQCRC2, SDHB and NDUFB8. (J, K) Relative mRNA levels of mitochondria-related genes in sh1 and sh2 after 6-day of differentiation. Data represent mean±s.e.m. (t-test: *P<0.05, **P<0.01).

**Figure S7. *Acss3* KO mice have reduced energy expenditure and are less active.**

(A-C) O_2_ consumption (left) measured by an indirect calorimetry is shown for a 36-hour cycle, and average day and night O_2_ consumption (VO_2_, right) of 3-month-old WT and Acss3^-/-^ mice as corrected to body weight (A), lean mass (B) and Fat mass (C). N=5 pairs of mice. (D) Average food intake of WT and Acss3^-/-^ mice, N=6 pairs of mice. (E) Locomotor activity of WT and Acss3^-/-^ mice as measured by voluntary wheel running, N=5 pairs of mice. Data represent mean±s.e.m. (t-test: **P<0.01).

**Figure S8. *Acss3* KO mice have minor change upon acute cold challenge/chronic cold treatment.**

(A) Rectal temperature of WT and Acss3^-/-^ mice during 6 h of acute cold challenge. N=6 pairs of mice. (B) Representative images of BAT and WAT depots from male mice showing reduced BAT mass and increased WAT mass of Acss3^-/-^ mice at room temperature (RT) or after 7-day of cold exposure (CT). (C) Weights of various BAT and WAT depots from male mice at RT and CT, N=4 pairs. (D) Relative levels of *Acss3*, *Acss1*, *Acss2*, *Ucp1* and *Pgc1α* (lower, N = 4), from WT and Acss3^-/-^ mice. UTR: a pair of primers detect the *3’ UTR* of *Acss3* mRNA. N=4 pairs of WT and Acss3^-/-^ mice. (E, F) H&E staining of BAT (E) and iWAT (F) from WT and Acss3^-/-^ mice after CT, scale bar: 50 μm. Data represent mean±s.e.m. (t-test: **P<0.01).

**Figure S9.** **Correlations of propionate and *ACSS3* expression to obese and insulin resistance in human.**

(A) Mean total SCFA concentrations in fecal samples of lean, overweight and obese individual, N=30, 35 and 33 for BMI at 18.5-24.9 (lean), 25-30 (overweight) and >30 (obese), respectively *(1)*. (B) Serum propionate concentrations from healthy individuals (N=35) and T2D patients (n=65) *(2)*. (C, D) *ACSS3* (C) *and LAMP1* (D) levels in WAT from lean and obese people *(3)*. (E) *ACSS3* expression levels in WAT from normal insulin tolerance and insulin resistant people *(4)*.

**Figure S10. Propionate treatment does not affect cell viability.**

Relative cell viability of human A41 preadipocytes after treated with 0.3- and 3 mM propionate for 24 hour, as indicated by crystal violet staining. N=3 independent treatments.

**Figure S11. HCQ/wortmannin treatment inhibits propionate-induced autophagy adipocytes.**

(A) Western-blot analysis (upper) of LC3 and P62 and ratios of LC3 II/I and P62/GAPDH (lower) of differentiated A41 white adipocytes treated with 0.3 mM propionate. (B) Western-blot analysis (upper) of LC3 and P62 and ratios of LC3 II/I and P62/GAPDH (lower) of differentiated A41 white adipocytes (left) and BAT cells treated with 3 mM propionate with/without wortmannin treatment. (C) Western-blot analysis of LC3 and P62 of differentiated mouse BAT cells treated with 3mM propionate with/without HCQ treatment. (B) Rapamycin treated cells was set as positive control. (D) Staining of P62 (green) of propionate treated BAT cells with/without HCQ treatment. (E) Western-blot analysis of LC3 and P62 of differentiated human A41 white adipocytes treated with 3mM propionate with/without HCQ treatment.  Data represent mean±s.e.m. (t-test: *P<0.05, **P<0.01).

**Figure S12. HCQ treatment inhibits autophagy and ameliorates hepatic steatosis in Acss3^–/–^ mice.**

(A) Western-blot analysis of LC3, P62, UCP1 and FABP4 in BAT of WT and Acss3^-/-^ mice with/without 0.15 mmol/L HCQ treatment after 10-week of HFD feeding. (B) Rapamycin treated cells was set as positive control. (B) Relative levels of *Atg5* in BAT. (C) Representative images of male WT and Acss3^-/-^ mice with/without 0.15 mmol/L HCQ treatment after 10-week of HFD feeding. (D, E) Representative images (D) and weights (E) of liver. N=4 pairs of male mice at starting at 2-month-old. Data represent mean ± s.e.m. (t-test: ** P<0.01).​

**Supplementary reference**

1. A. Schwiertz, D. Taras, K. Schäfer, S. Beijer, N. A. Bos, C. Donus, P. D. Hardt, Microbiota and SCFA in lean and overweight healthy subjects. *Obesity* **18**, 190-195 (2010).

2. L. Zhao, H. Lou, Y. Peng, S. Chen, L. Fan, X. Li, Elevated levels of circulating short-chain fatty acids and bile acids in type 2 diabetes are linked to gut barrier disruption and disordered gut microbiota. *Diabetes Research Clinical Practice* **169**, 108418 (2020).

3. Y. H. Lee, S. Nair, E. Rousseau, D. B. Allison, G. P. Page, P. A. Tataranni, C. Bogardus, P. A. Permana, Microarray profiling of isolated abdominal subcutaneous adipocytes from obese vs non-obese Pima Indians: increased expression of inflammation-related genes. *Diabetologia* **48**, 1776-1783 (2005).

4. O. T. Hardy, R. A. Perugini, S. M. Nicoloro, K. Gallagher-Dorval, V. Puri, J. Straubhaar, M. P. Czech, Body mass index-independent inflammation in omental adipose tissue associated with insulin resistance in morbid obesity. *Surgery for Obesity Related Diseases* **7**, 60-67 (2011).

**Table S1.**

Primers used in this study.

| Primer | Sequence (5’—3’) |
| --- | --- |

| **Genotyping PCR**  *Acss3*  **Real-time PCR**  *qAcss3*  *qUcp1*  *qPparγ*  *qC/ebpα*  *qAtg7*  *qAtg5*  *qBeclin1*  *qGabarap*  *qLamp1*  *qPgc-1α*  *qFabp4*  *qLc3b*  *qUlk1*  *qAtgl*  *qAcss1*  *qAcss2* | F: GCTTGCTGTCTTAGTTAGGGTTTC  R1: GAAGACACTCAAATCAACCTCAG  R2: CTTGCTGCCATAGATTTCCTTAG  F: AATGTCGCAAAGTAACAGGCG  R: GTGGGTCTTGTACTCACCACC  F: AGGCTTCCAGTACCATTAGGT  R: CTGAGTGAGGCAAAGCTGATTT  F: TCGCTGATGCACTGCCTATG  R: GAGAGGTCCACAGAGCTGATT  F: CAAGAACAGCAACGAGTACCG  R: GTCACTGGTCAACTCCAGCAC  F: GTGGGTCTTGTACTCACCACC  R: CCACTGAGGTTCACCATCCT  F: TAGAATATATCAGACCACGACG  R: CTCCTCTTCTCTCCATCTTC  F: GGCCAATAAGATGGGTCTGA  R: GCTGCACACAGTCCAGAAAA  F: GCTGCACACAGTCCAGAAAA  R: GTCCCGGTGATAGTGGAAAA  F: TAGTGCCCACATTCAGCATCTCCA  R: TTCCACAGACCCAAACCTGTCACT  F: TATGGAGTGACATAGAGTGTGCT  R: CCACTTCAATCCACCCAGAAAG  F: AAGGTGAAGAGCATCATAACCCT  R: TCACGCCTTTCATAACACATTCC  F: ACAAAGAGTGGAAGATGTCCGGCT  R: TGCAAGCGCCGTCTGATTATCTTG  F: AGATTGCTGACTTTGGATTC  R: AGCCATGTACATAGGAGAAC  F: CTGAGAATCACCATTCCCACATC  R: CACAGCATGTAAGGGGGAGA  F: GTTTGGGACACTCCTTACCATAC  R: AGGCAGTTGACAGACACATTC  F: AAACACGCTCAGGGAAAATCA  R: ACCGTAGATGTATCCCCCAGG |
| --- | --- |
